# Non‐Monotonic Variation of Potential‐Dependent Surface Diffusion at Electrochemical Interfaces in the Presence of Coadsorbates

**DOI:** 10.1002/anie.202419390

**Published:** 2025-02-11

**Authors:** Chaolong Yang, Reihaneh Amirbeigiarab, Sönke Buttenschön, Eckhard Pehlke, Olaf M. Magnussen

**Affiliations:** ^1^ Institut of Experimental and Applied Physics Kiel University Olshausenstr. 40 24098 Kiel Germany; ^2^ Institut für Theoretische Physik und Astrophysik Christian-Albrechts-Universität zu Kiel Olshausenstr. 40 24098 Kiel Germany

**Keywords:** surface diffusion, scanning tunnelling microscopy, non-monotonic potential dependence, density functional theory, Monte-Carlo simulations

## Abstract

The influence of coadsorbed ions on adsorbate diffusion, an inherent effect at solid–liquid interfaces, was studied for adsorbed sulfur on Ag(100) electrodes in the presence of bromide or iodide. Quantitative in situ high‐speed scanning tunnelling microscopy (video‐STM) measurements were performed both in the potential regime of the c(2×2) halide adlayer at its saturation coverage and in the regime of a disordered adlayer where the halide coverage increases with potential. These studies reveal a surprising non‐monotonic potential dependence of S_a*d*_ diffusion with an initial increase with halide coverage, followed by a decrease upon halide adlayer ordering into the c(2×2) structure. Density functional theory (DFT) and Monte Carlo (MC) simulations only qualitatively reproduce the rise in S_a*d*_ mobility with halide coverage, suggesting that many‐adsorbate interactions and the presence of the electrolyte need to be considered.

## Graphical abstract

Using fast scanning tunnelling microscopy measurements and complementary simulations, the diffusion of adsorbates at electrochemical interfaces is shown to depend in complex and unexpected ways on potential and coadsorbates. For the case of sulfur diffusion on halide‐covered Ag(100) electrodes, first a pronounced increase in sulfur mobility with increasing halide surface density is found, followed by a strong decrease at halide saturation coverage.

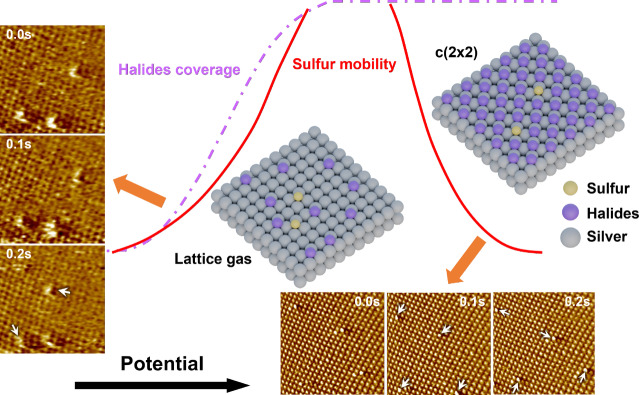

## Introduction

Surface diffusion is an important elemental step in many interface processes. While the diffusion of adsorbates on clean surfaces is well understood,^[1]^ much less is known about diffusion on surfaces that are crowded by a high coverage of another adsorbate species. Naively, one might assume that such coadsorbates block lattice sites and thus hinder the adsorbate's motion. However, recent studies of oxygen diffusion on a p(2×2)‐CO covered Ru(0001) surface by Henß et al. revealed that the O_a*d*_ diffusion rates were only marginally lower than in the absence of the CO adlayer.^[2]^ The unexpectedly high O_a*d*_ surface mobility was explained by a door‐opening mechanism, where the local density fluctuation in the co‐adsorbed layer promote the diffusion of the O atoms.

We here investigate the role of coadsorbates on diffusion at solid–liquid interfaces, where the presence of a high‐density coadsorbate adlayer is an inherent system property. These interfaces are of great current interest in electrochemical energy science as well as in wet‐chemical growth, corrosion, and self‐assembly processes. The presence of chemisorbed species, in particular halides, and the applied electrode potential are known to strongly affect the surface mobility in these systems.[^3^, ^4^]

This was shown most directly in studies by video‐STM by our group, where we quantitatively determined from observations of individual adsorbate site hopping events the tracer diffusion rates of sulfur (S_a*d*_), methyl thiolate (CH_3_S_a*d*_), and lead (Pb_a*d*_) on Ag(100) and Cu(100) electrode surfaces that were fully covered by ordered c(2×2) adlayers of chloride or bromide.[^4^, ^5^, ^6^, ^7^, ^8^, ^9^, ^10^, ^11^] In all cases, a strong linear change of the diffusion barrier $E_{d}$ with the potential Φ was found. It can be rationalized by an electrostatic energy contribution, resulting from the interaction of the adsorbate with the interfacial field and depending on the change in the surface dipole moment Δμ along the diffusion pathway to a neighboring site.[^3^, ^4^] In combination with DFT calculations, these studies revealed a very pronounced contribution of the surrounding halide coadsorbates to the barriers and dipole moment changes, which in extreme cases could even result in an inverted potential dependence.^[10]^

In all previous studies the influence of an ordered coadsorbate adlayer at saturation coverage was investigated. In contrast, adsorbate diffusion in the presence of a sub‐saturation adlayer of mobile coadsorbates has not been addressed, although this is a very common case in electrochemical systems. We focus on this case in the present work, where we study surface diffusion in the presence of a coadsorbate adlayer of variable coverage by in situ video‐STM and complementary combined DFT calculations and equilibrium MC simulations. As examples, we use sulfur diffusion on bromide or iodide covered Ag(100) electrodes. As shown by electrochemical studies,^[12]^ sulfur is strongly irreversibly adsorbed on Ag over the entire potential range used in this study.

In these systems, the halide anions initially adsorb in form of a highly mobile lattice gas. With increasing potential, the halide coverage increases until it undergoes a two‐dimensional Ising disorder‐order transition to a c(2×2) phase above a critical potential.[^13^, ^14^, ^15^] This type of adsorption behavior is prototypical for halides on fcc (100) surfaces and found in many systems.^[16]^ As we will show in the following, surprising differences in the S_a*d*_ diffusion within the disordered and the c(2×2) ordered coadsorbate adlayer are found.

## Results and Discussion

The video‐STM experiments were performed in electrolytes containing 1 mM bromide and iodide at room temperature (RT, ≈20 °C) as well as at 4 °C. Similar as in our previous video‐STM studies,[^4^, ^5^, ^6^, ^7^, ^8^, ^9^, ^10^, ^11^] small amounts of Na_2_S were introduced to the electrochemical cell, resulting in a potential‐independent coverage of irreversibly adsorbed S_a*d*_ in the range 1 to 10 %. Details of the experiments are provided in the Supporting Information.^[17]^ All potentials are given with respect to the saturated calomel electrode (SCE).

**Figure 1:**
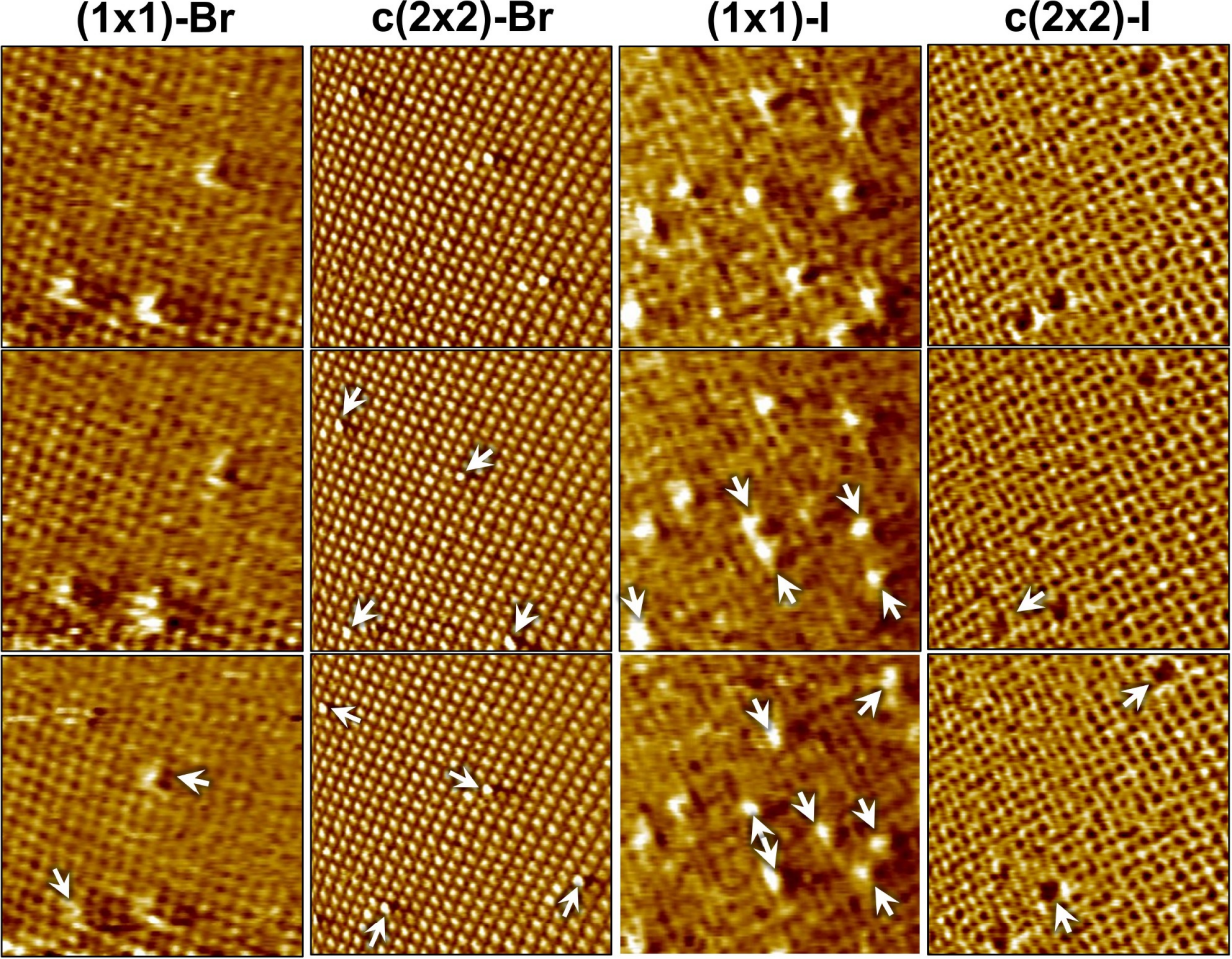
Representative in situ video‐STM sequences, showing S_a*d*_ diffusion in the presence of the (1×1) lattice gas and the c(2×2) adlayer of bromide and iodide, respectively. The images were recorded (from left to right) in 1 mM KBr+1 mM NaClO_4_ at −0.96 and −0.09 V and in 1 mM KI+1 mM NaClO_4_ and 1 mM KI+1 mM HClO_4_ at −1.38 and −0.22 V at time intervals of 0.1 s. Diffusion events are indicated by arrows.

Figure 1 shows characteristic video‐STM sequences of S_a*d*_ diffusion in the potential range of the lattice gas and of the c(2×2) covered surface. In the first case, the S_a*d*_ diffuse via hopping between sites of the Ag(100)‐(1×1) lattice. In the images, only a rather uniform (1×1) lattice and the S_a*d*_ are visible, indicating that the halide surface mobility in the lattice gas is too high to allow direct imaging by STM (>10^4^ s^−1^). In the potential range of the c(2×2) phase, the S_a*d*_ substitute halides in the ordered adlayer and diffuse on the superlattice, as previously reported for S_a*d*_ diffusion on Cl and Br covered Ag(100).^[8]^

**Figure 2:**
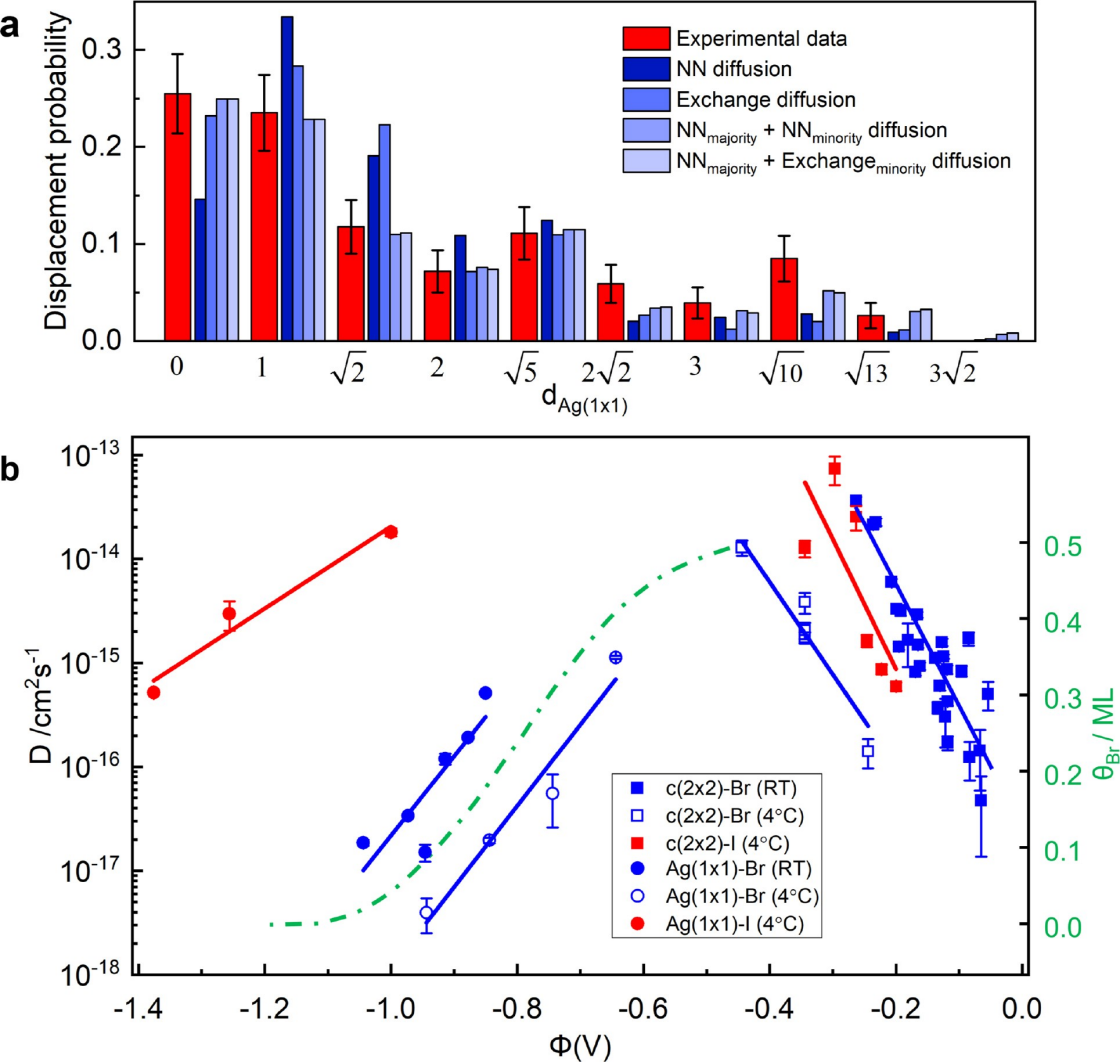
(a) Exemplary jump distribution function of the experimental data (−0.644 V, 4 °C) and best fits by 4 different models. (b) Potential‐dependent diffusion coefficients for the majority transport process of S_a*d*_ on the c(2×2) (squares) and (1×1) (circles) lattice in electrolytes containing 1 mM bromide (blue) or iodide (red) (data for S_a*d*_ diffusion on Ag(100)‐c(2×2)‐Br at RT are taken from Ref. [8]. Copyright 2018 Wiley‐VCH). The data on the c(2×2)‐halide surface were obtained from fits by the NN model, the data in the (1×1) regime correspond to the jump rate of the slow majority diffusion pathway in the NN_*majority*_+NN_*minority*_ diffusion model (the errors correspond to the range in which the least‐square deviation is ≤5 % from the optimum value). The dashed green line reproduces the Br_*ad*_ coverage in 1 mM Br‐solution at RT obtained from chronocoulometric data by Wandlowski et al. (data taken from Ref. [14]. Copyright 2001 Elsevier).

The tracer diffusion coefficients *D* of isolated S_a*d*_ were obtained from fits of the experimentally measured jump distribution functions (see Supporting Information, Section 2 for details). Fits by models assuming a single mechanism, e.g. hopping to nearest neighbor (NN) adsorption sites or vacancy‐assisted diffusion, provide reasonable fits only on the c(2×2) covered surface, whereas they deviate from the experimental data in the potential regime where the halide adlayer is disordered (Figure 2a). The necessity of a second diffusion process is in accordance with previous observations of an additional minority transport pathway via subsurface diffusion, where S_a*d*_ species temporarily move via fast diffusion in the Ag surface layer.^[9]^ Fits by models that include this second pathway in addition to NN hopping can reasonably describe the jump distribution functions (see Supporting Information, Section 2.3). According to the fit results, NN diffusion is the majority transport mechanism and exhibits a surprising non‐monotonic potential dependence, illustrated in Figure 2b. As noted before,^[8]^ the STM tip influences S_a*d*_ diffusion on halide‐covered Ag(100) more strongly than on Cu(100), resulting in some scatter in the obtained diffusion rates. Nevertheless, clear trends are discernible. In the c(2×2) potential regime, the S_a*d*_ mobility decreases towards positive potentials, in accordance with our previous data for diffusion on c(2×2)‐Br covered Ag(100).^[8]^ However, S_a*d*_ diffusion on the (1×1) lattice exhibits the opposite potential dependence, i.e., an increase of the jump rates with potential.

In the intermediate potential range, where the disorder‐order phase transition occurs, coexistence of small, dynamically fluctuating c(2×2) ordered and disordered halide domains is expected. Fluctuations between a (1×1) and c(2×2) lattice were indeed be observed in STM images collected in the disorder‐order transition regime (Supporting Information, Figure S11). Individual S_a*d*_ are still visible in the video‐STM images, indicating residence times >0.1 ms (i.e., *D*<10^−12^ cm^2^ s^−1^), but their surface mobility is too high for a quantitative analysis. Furthermore, the latter is problematic, because the S_a*d*_ may diffuse in both (1×1) and c(2×2) lattice during two STM images due to the fluctuations in the halide adlayer. To increase the experimentally accessible range, STM studies at 4 °C were carried out, which results in significantly lower jump rates. For experiments in iodide‐containing solutions, where the mobility is even higher than in Br‐containing solution, quantitative data were only obtainable at these low temperatures. Although the diffusion rates could not be obtained over the entire double layer potential range, the data clearly indicate that the mobility of the S_a*d*_ adsorbates goes through a maximum in the intermediate range. The results for the Br‐covered surface at 4 °C, where the inaccessible region is only 200 mV wide, imply that this maximum is close to the disorder‐order transition of the halide adlayer.

In both (1×1) and c(2×2) regions an approximately exponential potential dependence of the S_a*d*_ mobility was observed, indicating a linear relation between diffusion barrier $E_{d}\left(\Phi \right)$ and the potential. The latter was calculated from $\nu =\nu_{0}\cdot \exp\left(-E_{d}\left(\Phi \right)/k_{B}T\right)$ , assuming the same attempt frequency $\nu_{0}$ =2.35 ⋅ 10^12^ s^−1^ reported by Tansel et al. in a detailed temperature‐dependent study of S_a*d*_ diffusion on c(2×2)‐Cl covered Cu(100).^[4]^ For S_a*d*_ diffusion on the c(2×2) surface, $dE_{d}/d\Phi$ of 0.68 eV/V (Br‐covered Ag(100) at room temperature),^[8]^ 0.54 eV/V (Br‐covered Ag(100) at 4 °C), and 0.69 eV/V (I‐covered Ag(100) at 4 °C) are obtained. For S_a*d*_ diffusion on the Ag(100)‐(1×1), we obtain $dE_{d}/d\Phi$ values of −0.44 eV/V (Br‐covered Ag(100) at room temperature), −0.43 eV/V (Br‐covered Ag(100) at 4 °C), and −0.21 eV/V (I‐covered Ag(100) at 4 °C).

The potential‐dependence in the c(2×2) regime is in accordance with most other studied adsorbate/coadsorbate systems[^4^, ^5^, ^6^, ^7^, ^8^, ^9^, ^10^, ^11^] and can be rationalized by a rotation diffusion mechanism, in which the lattice positions of S_a*d*_ and three neighboring halide coadsorbates rotate around a common center.^[10]^ However, the surprising increase in the surface mobility with potential in the (1×1) regime is not clear yet. According to combined surface X‐ray scattering and chronocoulometric studies of Ag(100) in 1 mM Br‐containing electrolyte at room temperature, the Br coverage monotonically increases in the potential range −1.1 to −0.6 V (see Figure 2b).[^13^, ^14^] Thus, the S_a*d*_ mobility increases in parallel to the halide coverage, implying that the coadsorbates substantially accelerate the surface diffusion (up to several orders of magnitude). Although halide‐induced increases in surface mobility have been reported before, these were restricted to metal adatoms and could be explained by the formation of metal‐halide surface complexes.[^18^, ^19^, ^20^] In contrast to the latter case, adsorbed sulfide and halides interact repulsively and a strong enhancement of S_a*d*_ diffusion by coadsorbed halides is not expected.

To obtain insight into the origin of the potential dependence of the S_a*d*_ tracer diffusion at low halide coverage we have performed density functional calculations in combination with Monte Carlo simulations (for previous experiment‐based MC approach for CO/Cu(111) see Ref. [21]). First we address S_a*d*_ hollow‐bridge‐hollow jumps as a candidate for the observed majority diffusion mechanism. After that we will focus on S_a*d*_ jumps via a nearby Ag substrate vacancy, thereby addressing another transport mechanism. The Ag vacancy assisted hop, however, is not included in our statistical MC model, solely DFT data will be interpreted.

The MC simulations are based on a lattice gas Hamiltonian for the Br_*ad*_‐Br_*ad*_ interaction on Ag(100). Lattice‐gas models have successfully been utilized by many authors to investigate halide adsorption on single‐crystal electrodes and to determine properties like voltammetry,[^22^, ^23^] electrosorption valency and lateral adatom interaction energies by fitting to experiment[^24^, ^25^] or to DFT data.^[26]^ In particular, in the case of Br/Ag(100) lattice‐gas MC simulations have been able to reproduce the order‐disorder transition of the Br adlayer as a function of electrode potential.[^27^, ^28^, ^29^] For our MC model (explained in detail in the Supporting Information, Section 4) of the S/Br/Ag(100) lattice gas, S_a*d*_ diffusion is assumed to solely take place by a hop of the S_a*d*_ to a neighboring empty hollow site via a bridge site of the substrate lattice. Adsorption and desorption events combined with diffusion of the S^2−^ through the liquid are not considered, as S_a*d*_ is found to be strongly bound in experiment and the desorption probability is not expected to increase with sample voltage even when the S_a*d*_‐Br_*ad*_ interaction is considered. Individual S_a*d*_ hollow‐bridge‐hollow hopping rates are averaged over all configurations of the Br_*ad*_ coadsorbate at a given Br chemical potential. The average S_a*d*_ hopping rate can readily be derived from equilibrium grand canonical Monte Carlo simulations of the Br coverage, given the lateral Br−Br and S−Br interaction energies from our DFT calculations. The result does not rely on the specific dynamics of the Br atoms (i.e. surface diffusion or adsorption‐desorption). The adatom interactions are approximated by a superposition of pair interactions. For the density functional calculations, we have applied the program package quantum espresso[^30^, ^31^] (see Supporting Information, Section 3). In Ref. [26] Juwono et al. parameterized the Br–Br interaction energy on Ag(100) by fitting their DFT data with a dipole‐dipole interaction. For the S−Br interaction we also identified the dipole‐dipole interaction as the most prominent contribution beyond next nearest neighbor separation, but due to the high accuracy required by our MC simulations other interaction mechanisms must not be neglected. To avoid inaccuracies due to some extrapolation of the interaction energies, we have chosen the size of the MC simulation cell equal to p(6×6), the size of the surface unit cell in the density functional calculations, and make direct use of the DFT data in the MC simulations. The configuration with S and a single Br in NN hollow position is not stable and therefore required stabilization of the Br adatom by additional Br adatoms (see Supporting Information, Section 3.2). This procedure is not critical since the MC results are insensitive to the precise values of these large NN adatom interaction energies.

**Figure 3:**
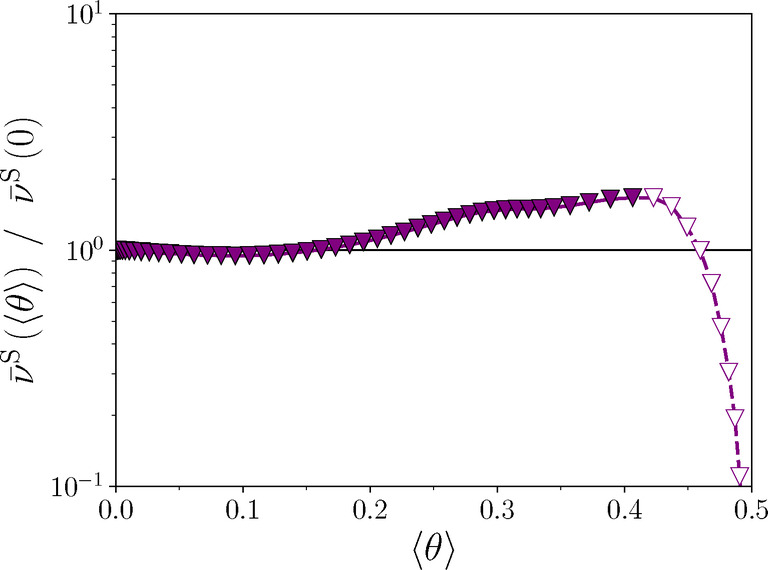
MC result for the average S hollow‐bridge‐hollow hopping rate ${\bar{\nu }}^{S}$ wrt. the hopping rate on the clean surface as a function of Br coverage. Open symbols indicate the coverage range in which more complex S diffusion paths with concerted S and Br motion neglected in the lattice gas approach may become relevant. Note the logarithmic scale on the ordinate.

Our MC simulations result in only a slight overall increase of the S hollow‐bridge‐hollow hopping rate with halogen coverage in the unordered (1×1) phase by a factor of about 1.8, with a maximum close to the order‐disorder transition (see Figure 3 and Supporting Information, Section 4.2). This is to be compared to the jump rate of the majority process observed in experiment, which increases by nearly two orders of magnitude. Thus, there is a clear quantitative discrepancy between the sample potential dependence from the MC model and the experimental findings. Moreover, the electric field effect, i.e. the increase of the diffusion energy barrier of the partially negatively charged S_a*d*_ with more positive sample potential,^[3]^ is not included in our MC model. The computed dipole moment change along the S diffusion path on the clean substrate amounts to −128 me Å. If the electric field effect were added, it would decrease any positive slope of the S_a*d*_ hollow‐bridge‐hollow hopping rate as a function of Br coverage.

To improve our results for the S hollow‐bridge‐hollow hopping rate, many‐adsorbate interaction effects and atomic relaxation of the Br adlattice as in Ref. [32] should be incorporated in the MC simulations. In Ref. [33] it has been shown that lateral adsorbate interaction effects going beyond pair superposition can have strong impact on the tracer diffusion coefficient.

Motivated by the experimental indication for a second S_a*d*_ diffusion mechanism, and in view of prior experimental evidence for subsurface S atom diffusion in case of the fully c(2×2)‐Br‐covered Ag(100) surface,^[9]^ we have examined additional diffusion paths in which S_a*d*_ hops into an Ag surface vacancy in the top layer of the Ag substrate. These jumps have been investigated with DFT for various configurations of the Br coadsorbates. In case of low Br coverages (i.e. in the unordered halogen adlayer regime) the S atom residing in an Ag vacancy is energetically unfavorable compared to the S atom adsorbed on the surface. This suggests an S_a*d*_ diffusion process via hopping into and out of a NN Ag vacancy, by which the S adatom can, in some cases, cover a lateral distance longer than one p(1×1) lattice spacing. To compare the probabilities of S_a*d*_ hollow‐bridge‐hollow hops and S‐into‐vacancy hops, the energy required for the formation of an Ag vacancy NN to the S_a*d*_ (which depends on the particular S−Br configuration) has to be accounted for. It governs the Ag vacancy concentration close to S_a*d*_ on the surface.^[34]^ Furthermore we include the statistical weight of the S−Br configuration by adding the lateral Br−Br and S−Br interaction energies to the activation energies of the S_a*d*_ hollow‐bridge‐hollow hops and the S‐into‐vacancy hops. Details are explained in the Supporting Information,^[17]^ Section 5. The hollow‐bridge‐hollow hops are treated statistically on the basis of our MC model. Due to the lack of a simple MC model describing the Ag vacancy formation together with the S_a*d*_ vacancy‐assisted diffusion, we compare a large sample of S‐hopping‐into‐Ag‐vacancy paths (comprising different Br coadsorbate configurations) with the statistical average for the hollow‐bridge‐hollow paths from the MC simulations. The sample has been chosen by hand with some preference for S−Br configurations with small S−Br and Br−Br interaction energy. Our comparison between both types of S_a*d*_ diffusion paths does not show any clear energy preference towards either of them, indicating that both might contribute to the sulfur hopping rate observed in experiment. Examination of the S‐into‐vacancy paths reveals a dipole moment change along the diffusion path which is of similar magnitude but opposite in sign compared to the hollow‐bridge‐hollow hop, thus predicting a rise of the respective S_a*d*_ hopping rate with sample potential due to the aforementioned electric field effect. However, in case of the secondary sulfur diffusion process no distinct potential dependence has been observed in experiment, thus pointing out another discrepancy between our experimental and theoretical results.

## Conclusion

In conclusion, a different sign of the change in jump rate with potential has been observed in video‐STM measurements for S_a*d*_ tracer diffusion on Ag(100) surfaces at low and high coverage with Br coadsorbates. Theoretical combined MC and DFT results for the surface versus vacuum cannot resolve the astounding sample potential dependence of the S hopping rate within the unordered phase of the Br coadsorbate. While for the direct hollow‐bridge‐hollow jump we obtain a slight increase of hopping rate with Br coverage, it has turned out to be much too small in comparison to experiment, which we attribute to limitations in the modeling. For example, many‐adsorbate interactions are not included in the present MC simulations. These could be directly inferred from DFT calculations. Furthermore, the presence of the electrolyte was not taken into account. The discrepancy between the theoretical results *in vacuo* and the experimental results in electrochemical environment might suggest a profound influence of the latter on the sulfur tracer diffusion. To examine this, future DFT simulations could include explicit water molecules.[^35^, ^36^]

## Conflict of Interests

The authors declare no conflict of interest.

1

## Supporting information

As a service to our authors and readers, this journal provides supporting information supplied by the authors. Such materials are peer reviewed and may be re‐organized for online delivery, but are not copy‐edited or typeset. Technical support issues arising from supporting information (other than missing files) should be addressed to the authors.

Supporting Information

## Data Availability

The data that support the findings of this study are available from the corresponding author upon reasonable request.
