# Supplementary material for: Non‐Monotonic Variation of Potential‐Dependent Surface Diffusion at Electrochemical Interfaces in the Presence of Coadsorbates
Source: Angew Chem Int Ed Engl. 2025 Feb 11;64(10):e202419390. doi: 10.1002/anie.202419390 (PMC11878336; doi:10.1002/anie.202419390)
Supplement: Supplementary file 1 — Supporting Information [file ANIE-64-e202419390-s001.pdf]

Angewandte Chemie
Eine Zeitschrift der Gesellschaft Deutscher Chemiker

Supporting Information

Non-Monotonic Variation of Potential-Dependent Surface Diffusion at Electrochemical Interfaces in the Presence of Coadsorbates

C. Yang, R. Amirbeigiarab, S. Buttenschön, E. Pehlke, O. M. Magnussen**

Supporting Information for

“Non-monotonic variation of potential-dependent surface diffusion at electrochemical interfaces in the presence of coadsorbates"

Chaolong Yang, Reihaneh Amirbeigiarab, Sönke Buttenschön, Eckhard Pehlke, Olaf M. Magnussen

Contents

1. Experimental details **3**

2. Quantitative data analysis **3**
2.1. Determination of experimental jump distribution functions . . . 3
2.2. Determination of diffusion coefficients . . . 3
2.2.1. General procedure . . . 3
2.2.2. Vacancy-assisted diffusion . . . 4
2.2.3. Transport via two diffusion mechanisms . . . 5
2.3. Comparison of the results by different diffusion models . . . 6

3. Details on the density functional calculations **10**
3.1. Computational details . . . 10
3.2. Br–Br and S–Br interaction energy on Ag(100) . . . 11
3.2.1. Definition of the interaction energy . . . 11
3.2.2. Adatom configurations . . . 11
3.2.3. Accuracy of the DFT interaction energy . . . 16

4. Details on the lattice gas model **16**
4.1. Derivation of the average S_{ad} hollow-bridge-hollow hopping rate . . . 16
4.2. Details on the Monte Carlo simulations . . . 18

5. Details on S diffusion via substrate vacancy **21**

1. Experimental details

A home-built electrochemical Video-STM with a high temporal resolution (in the range of 0.1 s) has been used to carry out the *in situ* experiments, which allows a direct observation of the dynamic processes at the interface.[1,2] The electrochemical cell of this STM consists of a Teflon ring of 8 mm diameter and 3 mm height, pressed against the Ag(100) single electrode surface (MaTeck, 10mm Diameter), which forms the bottom of the cell. An Ag/AgCl reference electrode and a platinum wire served to determine the cell potential. The experiments were carried out at 4 °C and under room temperature condition. Ag(100) single crystals (MaTeck) were prepared by a chemical polishing procedure as described in[3], followed by inductive annealing at ≈ 750 °C under argon atmosphere. The following electrolytes have been used: 1 mM $HClO_4$ + 1 mM KBr, 1mM $NaClO_4$ + 1mM KBr, 1 mM KBr, 1 mM $HClO_4$ + 1 mM KI and 1 mM $NaClO_4$ + 1 mM KI. All electrolytes have been prepared from high-purity $HClO_4$ (Merck, suprapure), KBr (Merck, p.a.), KI (Merck, p.a.), $NaClO_4$ (Sigma Aldrich) and ultrapure water (18.2 MΩcm). The samples were immersed in bromide/iodine free solutions to avoid the AgBr/AgI bulk formation, after establishing the potential control in the electrochemical cell, the electrolyte (depth≈2mm, volume≈100μL) has been exchanged at the double layer potential region by bromide/iodine containing solution. Subsequently, 2 to 10 μl of a 5 to 10 μM Na_2S (Alfa Aesa, p.a.) was added to the cell, resulting in a total amount of S^{2-} in the electrolyte of 10^{-11} to 10^{-10} Mol. After insertion of the sulfide ions about 45 minutes were allowed before approaching the STM tip and starting the measurements. Because of the small depth of the electrolyte layer on top of the sample, this waiting time is enough for the diffusion and irreversible adsorption on the Ag surface of all sulfur ions in the electrolyte. The dosing procedure results in sulfur coverages <5%, which is in good agreement with the values expected for complete adsorption of all S^{2-} (considering that the exposed electrode area corresponds to ≈10^{-9} Mol Ag surface atoms). The same procedures were carried in our previous Video-STM studies of surface diffusion and the resulting sulfur coverage (estimated from the STM images) was shown to change approximately linearly with the amount of sulfide added [5], demonstrating the feasibility of controlled S_{ad} dosing.

2. Quantitative data analysis

2.1. Determination of experimental jump distribution functions

As a first step in the quantitative determination of the S_{ad} hopping rates on the Ag(100) surface, the motion of the individual adsorbates was determined for each video-STM frame using a home-built image recognition software, which generates a binary map for each frame of the video sequence marking the S_{ad} position on the detected lattice[4]. Fig. S1 shows three successive STM images and the corresponding generated binary maps to illustrate the method used for identifying the S_{ad} positions (blue and black dots in Fig. S1b) and quantifying its hopping rates.

After marking the adsorbate positions for each image in a video sequence, a distribution function is then generated for the jump rates of S_{ad}, which only considers adsorbates with a minimum distance of four lattice spacing to all neighboring adsorbates. Based on a detailed analysis of the S_{ad}–S_{ad} interaction in a reasonably comparable system (S_{ad}/c(2×2)-Cl-Cu(100)), it is well known that the S_{ad}–S_{ad} interaction has no major effect on the hopping rates at this distance and can be neglected[2,5]. The experimental errors were calculated via $\sqrt{N_i}$, where N_i is the number of experimental jumps to site i.

2.2. Determination of diffusion coefficients

2.2.1. General procedure

For a quantitative analysis of the S_{ad} surface transport, the experimental jump distribution functions were fitted by different continuous time random walk diffusion models, using a non-linear least square fitting algorithm (Quasi-Newton method[6]). To avoid that the fits are dominated by the jumps with low displacement probability, which have high experimental errors, the least-square deviations (LSD) rather than the χ^2 were used as optimization criteria. Errors of the fit parameters were obtained by determining the range, in which the LSD was ≤ 5% of the optimum value. Initially, a simple random walk on a square lattice with statistically independent jumps to neighboring sites at rate ν[7,8] was used, as employed in our previous studies of similar systems[2,9–13]. However, the best fits by this model significantly deviated from the experimental data. For this reason, more complex diffusion models were developed, which are described in the following. From the hopping rates ν of the best fit, the diffusion coefficients $D = \frac{1}{4}\nu_d a^2$ were calculated (with jump lengths a of 2.89 and 4.09 Å, respectively) for all studied systems to enable comparison of the diffusion on (1×1) and c(2×2)-covered Ag(100).

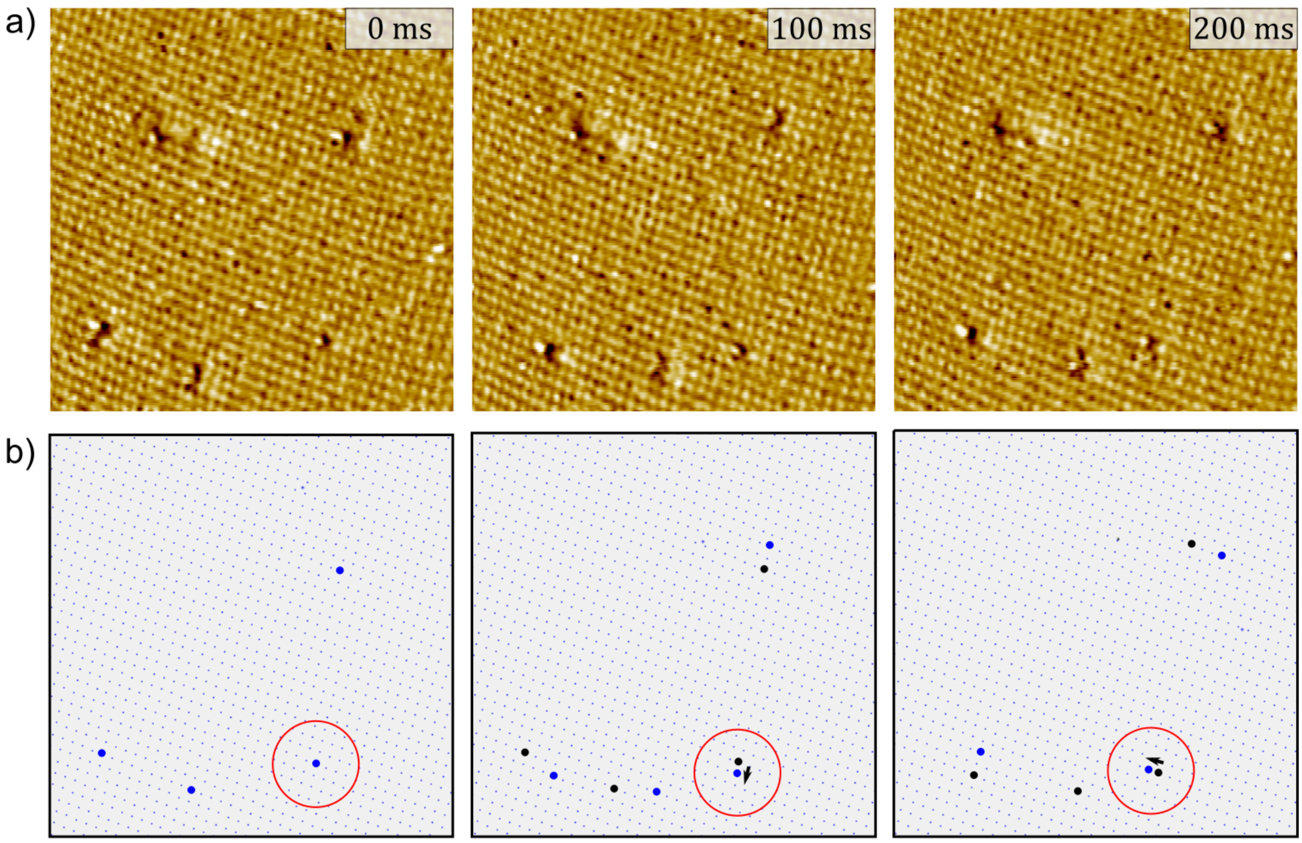

Figure S1. Representative example of the method used for the determination of the S_{ad} hopping rate. (a) STM images of S_{ad}/c(2×2)-I-Ag(100) at -0.24 V, recorded with a time resolution of 0.1 s. The dark single minima represent the tracer adsorbate. (b) The corresponding binary map of the iodine lattice. Blue and black dots marked the positions of adatoms on the respective and previous frame, respectively. The red circle illustrates the minimum S_{ad}–S_{ad} distance, for which the jump of each adsorbate can be considered as an independent event.

2.2.2. Vacancy-assisted diffusion

In vacancy-assisted diffusion the adsorbed species moves into an adjacent surface vacancy and then exits this vacancy again, with equal probability to each of the neighboring sites on the surface (Fig. S2). For a given position of the vacancy, this results in jumps to two of the nearest neighbor (NN) sites, to one of the next nearest neighbor (NNN) lattice sites, and to jumps back to the original position. Consequently, diffusion occurs by parallel jumps to NN and NNN sites, with the probability for direct jumps to NNN sites being half of the probability for jumps to NN sites. As jump distribution functions for parallel NN and NNN diffusion on a square lattice have not been published yet to our knowledge, we here derive the latter explicitly.

We consider a more general model, where we assume nearest neighbor (NN) jumps and next nearest neighbor (NNN) jumps in one direction take place at two different rates a and b, respectively (Fig. S2). This describes a situation where in each elementary diffusion step the adsorbate moves either by hopping on the surface or by vacancy-assisted exchange. The corresponding Kolmogoroff equation [14], which specifies the probability $p_{x,y}$ that the atom is at the coordinates x, y expressed in units of the nearest neighbor spacing, is then given by:

$$\frac{dp_{x,y}}{dt} = a(p_{x+1,y} + p_{x-1,y} + p_{x,y+1} + p_{x,y-1}) + b(p_{x+1,y+1} + p_{x+1,y-1} + p_{x-1,y+1} + p_{x-1,y-1}) - 4ap_{x,y} - 4bp_{x,y}$$

Considering the probability generating function:

$$G(t, z, \zeta) = \sum_{x=-\infty}^{\infty} \sum_{y=-\infty}^{\infty} z^x \zeta^y p_{x,y}$$

where z and ζ are variables, the differential equation for the generating function is:

$$\dot{G} = \sum_{x=-\infty}^{\infty} \sum_{y=-\infty}^{\infty} z^x \zeta^y [a(p_{x+1,y} + p_{x-1,y} + p_{x,y+1} + p_{x,y-1}) + b(p_{x+1,y+1} + p_{x+1,y-1} + p_{x-1,y+1} + p_{x-1,y-1}) - 4ap_{x,y} - 4bp_{x,y}]$$

$$= G[a(\frac{1}{z} + z + \frac{1}{\zeta} + \zeta) + b(\frac{1}{z\zeta} + z\frac{1}{\zeta} + \frac{1}{z}\zeta + z\zeta) - 4a - 4b]$$

If at time $t = 0$ the atom always starts at $x = y = 0$, so that $G(0, z, \zeta) = 1$, therefore:

$$G(t, z, \zeta) = \mathrm{e}^{at[(\frac{1}{z}+z+\frac{1}{\zeta}+\zeta)-4]+bt[(\frac{1}{z\zeta}+z\frac{1}{\zeta}+\frac{1}{z}\zeta+z\zeta)-4]}$$

Using Schlomilch's series:

$$\mathrm{e}^{\frac{\tau}{2}(z+\frac{1}{z})} = \sum_{n=-\infty}^{\infty} z^n I_n(\tau)$$

were $I_n(\tau)$ is the modified Bessel function of the first kind, the generating function can be written as:

$$\begin{aligned} G(t, z, \zeta) &= \mathrm{e}^{[-4(a+b)t]} \sum_{i=-\infty}^{\infty} z^i I_i(2at) \sum_{j=-\infty}^{\infty} \zeta^j I_j(2at) \sum_{k=-\infty}^{\infty} (z\zeta)^k I_k(2bt) \sum_{l=-\infty}^{\infty} (\frac{z}{\zeta})^l I_l(2bt) \\ &= \mathrm{e}^{[-4(a+b)t]} \sum_{i=-\infty}^{\infty} \sum_{j=-\infty}^{\infty} \sum_{k=-\infty}^{\infty} \sum_{l=-\infty}^{\infty} z^{i+k+l} \zeta^{j+k-l} I_i(2at) I_j(2at) I_k(2bt) I_l(2bt) \end{aligned}$$

Employing the substitutions $x = i+k+l$ and $y = j+k-l$, the distribution of the displacements can be extracted:

$$p_{x,y}(x, y, a, b, t) = \mathrm{e}^{-4(a+b)t} \sum_{k=-\infty}^{\infty} \sum_{l=-\infty}^{\infty} I_{x-k-l}(2at) I_{y-k+l}(2at) I_k(2bt) I_l(2bt)$$

For the 2D random walk model with jumps to NN sites, $b = 0$ and the only fit parameter for the distribution function P is a:

$$P = p_{x,y}(x, y, a, 0, t) \tag{1}$$

For the model including vacancy-assisted diffusion, where we consider direct jumps to NN and NNN sites, the fitting parameters are two independent rates a and b:

$$P = p_{x,y}(x, y, a, b, t) \tag{2}$$

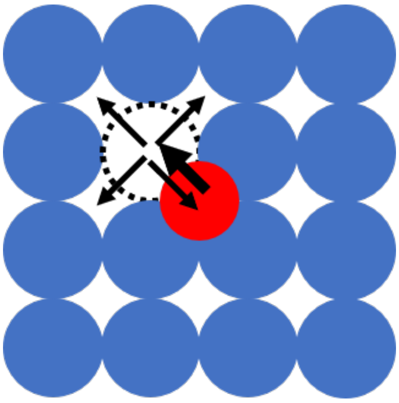

Figure S2. Schematic diagrams of vacancy-assisted diffusion, involving direct jumps to nearest and next nearest neighbor sites.

2.2.3. Transport via two diffusion mechanisms

In previous studies of S_{ad} diffusion on Ag(100) covered by a c(2×2) Br adlayer, we observed in addition to conventional hopping between NN sites a second transport mechanism, where the S_{ad} species moved 1-4 times per minute into a surface vacancy, then travelled for several hundred milliseconds by fast diffusion within the Ag surface layer, and then moved back onto the surface[15]. To approximately describe this behavior, we assume that in the time interval Δt between two subsequent Video-STM images the adsorbate either moves exclusively by on-surface hopping with rate a or exclusively by a second, faster process with the rate b (Fig. S3). The fraction of position changes via the first process is h, the fraction of the second process is $1 - h$ (with $0 \leq h \leq 1$). As will be shown later on, $h > 0.5$, i.e., the slow diffusion with rate a is the majority and the faster diffusion with rate b the minority process. In this model the total distribution function is:

$$P = h \cdot p_{x,y}(x, y, a, 0, t) + (1 - h) \cdot p_{x,y}(x, y, b, 0, t) \tag{3}$$

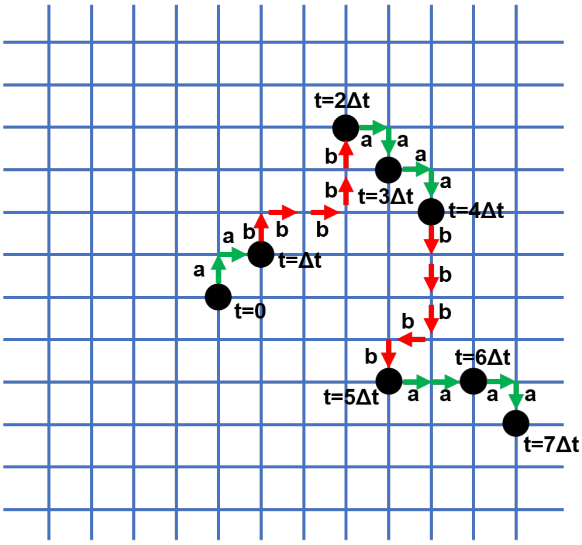

Figure S3. Schematic illustration of a transport process where the adsorbate switches between slow (on surface) diffusion by rate a and fast (sub-surface) diffusion by rate b.

In a variation of the above model, we assume that the fast minority diffusion process proceeds via the vacancy-assisted diffusion mechanism described in section 2.2.2, rather than by simple hopping between NN sites. For ideal vacancy-assisted diffusion, elementary jumps to NN sites with rate $2b$ and to NNN sites with rate b occur. Hence, the total distribution function is given by:

$$P = h \cdot p_{x,y}(x, y, a, 0, t) + (1 - h) \cdot p_{x,y}(x, y, 2b, b, t) \quad (4)$$

We also tested similar models where the majority process is vacancy-assisted diffusion, but these gave poorer fit results and are therefore not shown.

2.3. Comparison of the results by different diffusion models

All experimental data were fitted by the diffusion models described above. To compare the quality of the fits obtained by the different models we employed the least-square deviations LSD $= \sum (O_i - E_i)^2$ and:

$$\chi^2 = \sum \frac{(O_i - E_i)^2}{E_i}$$

where O_i are the observed values (experimental data) and E_i are the expected values given by the fit. To compare fitting models with different number of parameters, the F-test was used:

$$F_{2,1} = \frac{\frac{LSD_1 - LSD_2}{p_2 - p_1}}{\frac{LSD_2}{n - p_2}} = \frac{LSD_1 - LSD_2}{LSD_2} \cdot \frac{n - p_2}{p_2 - p_1}$$

Here, n is the number of datasets, p_1 and p_2 are the number of parameters involved in the compared models 1 and 2 (with $p_1 < p_2$), and LSD_1 and LSD_2 are the corresponding least-square deviations. A relatively large F (compared to some desired false-rejection probability of the F-distribution) suggests that model 2 gives a significantly better fit to the data than model 1.
Exemplary jump distribution function plots (Fig. S4-S7) show that on the (1×1) lattice, i.e., in the presence of the disordered halide adlayer, models where the S_{ad} alternate between two diffusion mechanisms (section 2.2.3) provide superior fits. In contrast to simple hopping diffusion to NN sites or vacancy-assisted diffusion, these 2-mechanisms models can describe the measured data within the experimental errors. In particular, they are capable to describe the long tail of the distribution function towards high values of d. However, this difference of the models can only be distinguished at relatively high diffusion rates, where multiple jump lengths are experimentally observed. In the regime of low rates, jumps of the S_{ad} to sites beyond NN sites are almost never observed (Fig. S8) and such have high experimental errors. Obviously, distinguishing between the different models is difficult for these data as differences to simple NN diffusion model only manifest in the probabilities for $d > 1$. These qualitative observations are confirmed by the LSD, χ^2 and F-tests (partial) for the data sets shown in Fig. S4 to S8 (Table S1). The vacancy-assisted diffusion model provides significantly lower LSD and χ^2 values than NN diffusion. However, even lower LSD and χ^2 values are found for the 2-mechanisms models. The F-tests also

indicate that the 2-mechanism models provide significantly better fits. In contrast, for the transport processes of S_{ad} on the c(2×2)-Br covered Ag(100) surface, the 2-mechanisms models do not fit the data better than hopping or vacancy-assisted diffusion, as illustrated in Fig. S9 and the corresponding χ^2 and F-tests (Table S1, last entry). This may suggest that the majority diffusion process is much more dominant in the presence of the ordered Br adlayer.

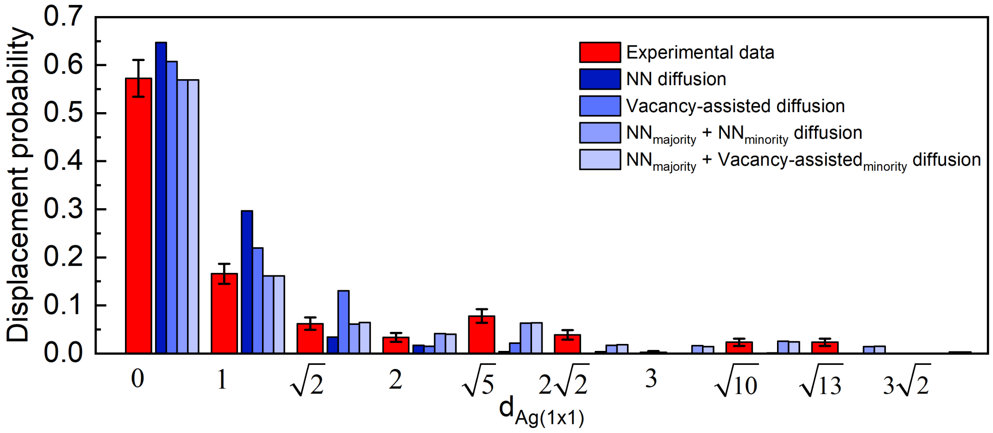

Figure S4. Exemplary jump distribution function of the experimental data at -0.85 V and room temperature (RT) and best fits by 4 different models.

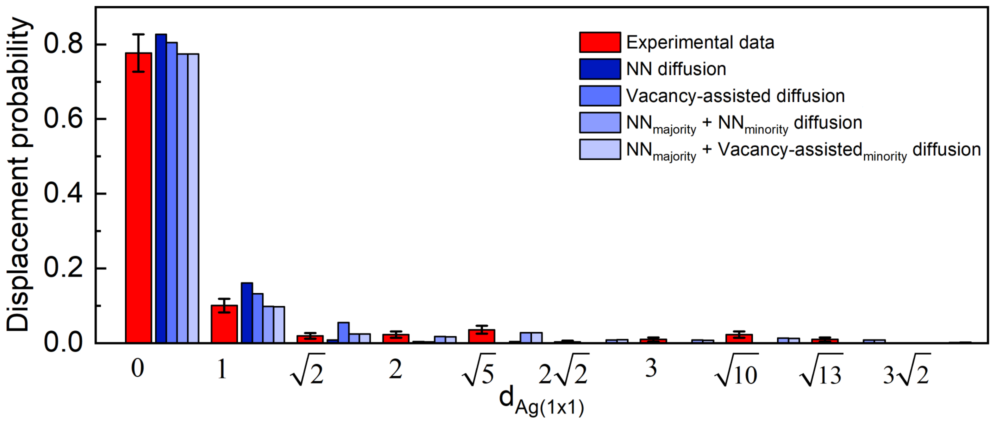

Figure S5. Exemplary jump distribution function of the experimental data at -0.88 V (RT) and best fits by 4 different models.

Table S1. Least-square deviations and χ^2 values of the best fits by 4 different models for the exemplary data sets as well as and F-tests for comparing the quality of the different models.

Pot./temp.	LSD_1	χ_1^2	LSD_2	χ_2^2	LSD_3	χ_3^2	LSD_4	χ_4^2	$F_{2,1}$	$F_{3,1}$	$F_{3,2}$
-0.85 V / RT	0.032	81.1	0.014	2.0	0.0011	0.053	9.1E-4	0.045	9.5	102.4	89.7
-0.88 V / RT	0.0086	706.6	0.0051	9.7	2.5E-4	0.018	2.7E-4	0.019	5.5	117.6	136.2
-0.95 V / RT	0.011	842.8	0.0033	4.0	1.4E-4	0.008	1.7E-4	0.012	18.4	276.2	162.4
-0.64 V / 4 °C	0.034	0.38	0.020	0.39	0.0020	0.051	0.0036	0.069	5.4	54.4	62.1
-0.74 V / 4 °C	0.0017	35.8	1.8E-4	0.399	5.8E-5	0.034	1.3E-5	0.007	70.9	102.0	14.4
-0.84 V / 4 °C	1.3E-5	0.316	7.6E-6	0.315	4.0E-6	0.006	5.5E-6	0.016	6.1	8.0	6.1
-0.26 V / RT	0.0041	0.073	0.0038	0.079	0.0036	0.067	0.0038	0.079	0.46	0.52	0.61

Fig. S10 shows the potential dependence of the fitting parameters h, a and b for the two 2-mechanisms models. Although h decreases towards positive potentials, it is always larger than 0.5 in the measured potential range, which indicates that slow NN diffusion is the majority transport mechanism. More importantly, the results indicate a clear trend for the potential dependence of the majority diffusion process, namely that the S_{ad} mobility

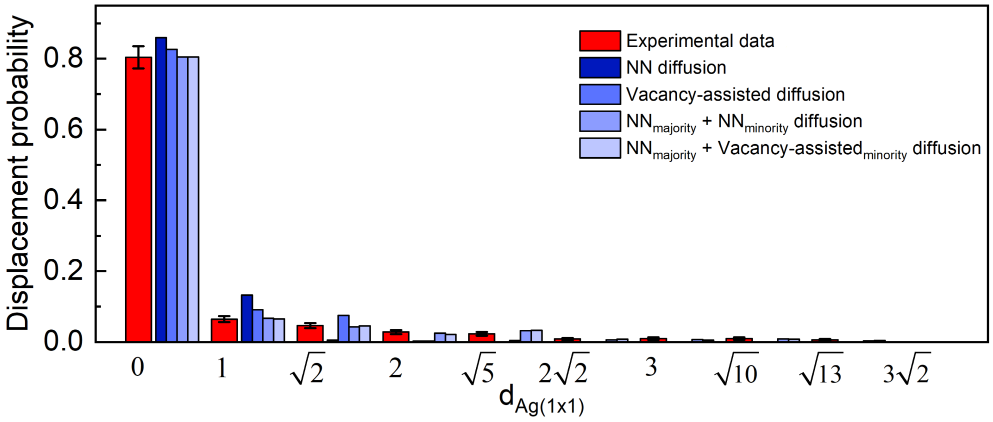

Figure S6. Exemplary jump distribution function of the experimental data at -0.95 V (RT) and best fits by 4 different models.

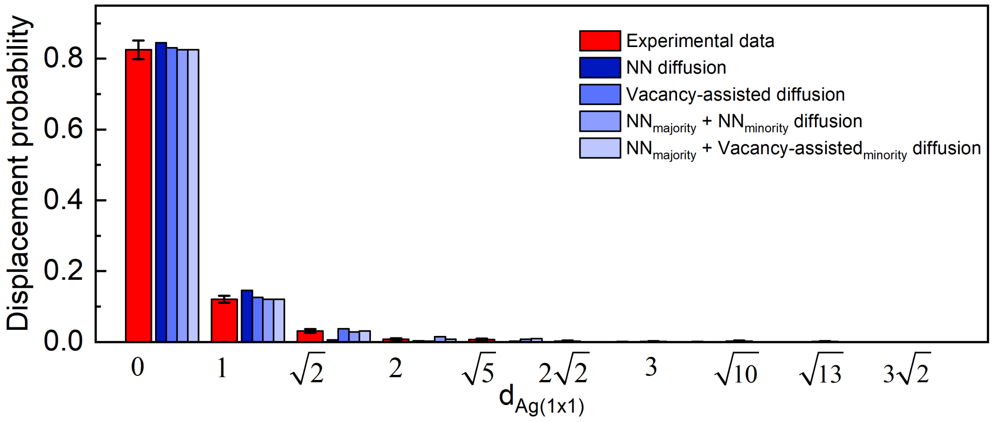

Figure S7. Exemplary jump distribution function of the experimental data at -0.74 V (4 °C) and best fits by 4 different models.

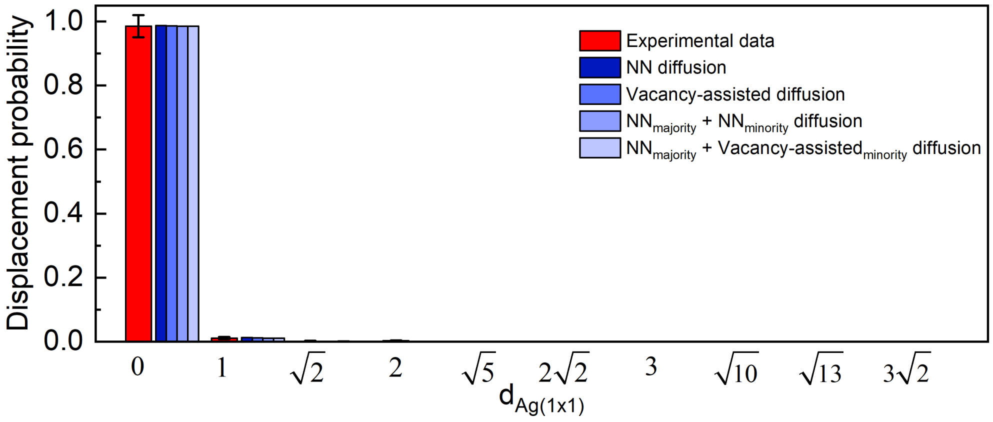

Figure S8. Exemplary jump distribution function of the experimental data at -0.84 V (4 °C) and best fits by 4 different models.

$4a$ (indicating the total probability for jumps in any of the 4 symmetrically equivalent directions) increases with increasing potential. This trend is found for both models with similar values of $4a$. In addition, the mobility decreases by approximately one order of magnitude upon changing the temperature from room temperature to 4 °C. The rates $4b$ of the minority process only shows a temperature effect. The potential dependence does not exhibit clear trends, which may be caused by the involvement of surface vacancies in the minority process. The density of surface vacancies depends on the local surface topography, specifically the distance of the measured area to adjacent atomic steps, and may thus differ in the different Video-STM measurements. Together with

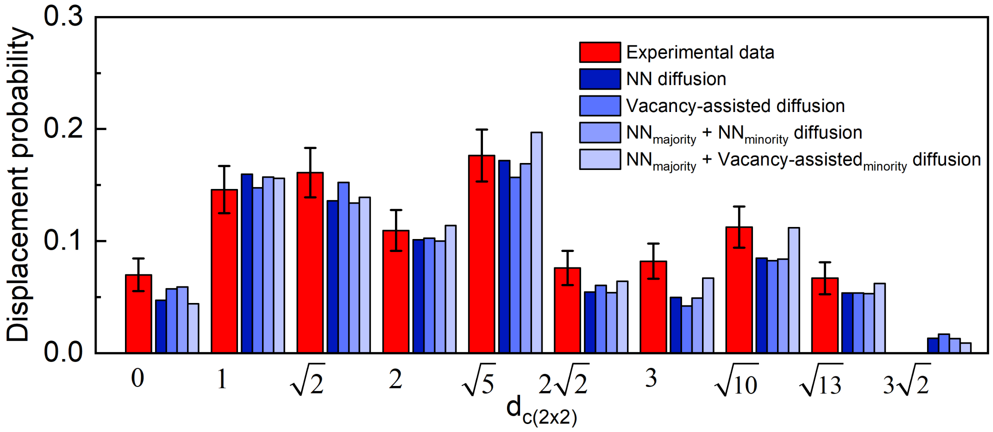

Figure S9. Exemplary jump distribution function of the experimental data at -0.26 V (RT) and best fits by 4 different models.

the limited statistics, these differences may result in systematic deviations between the rates obtained from the individual video sequences and thus hamper a precise quantitative analysis.

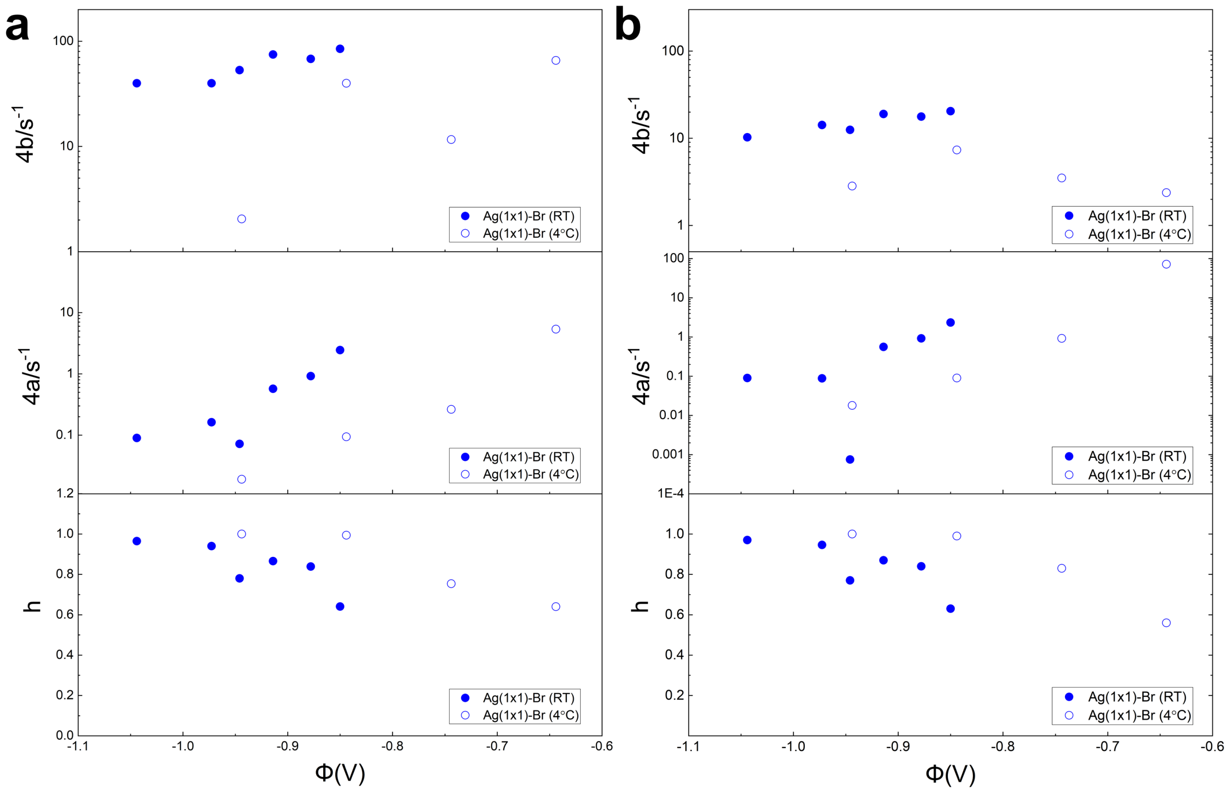

Figure S10. Fitting parameters h, a, b versus potential for the 2-mechanism models with (a) two NN diffusion processes and (b) NN and vacancy-assisted diffusion of S_{ad} on the Ag(1×1) lattice. The data at -0.97 V was obtained by averaging 3 (short) videos, to improve the statistics.

Fig. S11 shows the dynamically fluctuating c(2x2) ordered and disordered halide domains in the intermediate potential range.

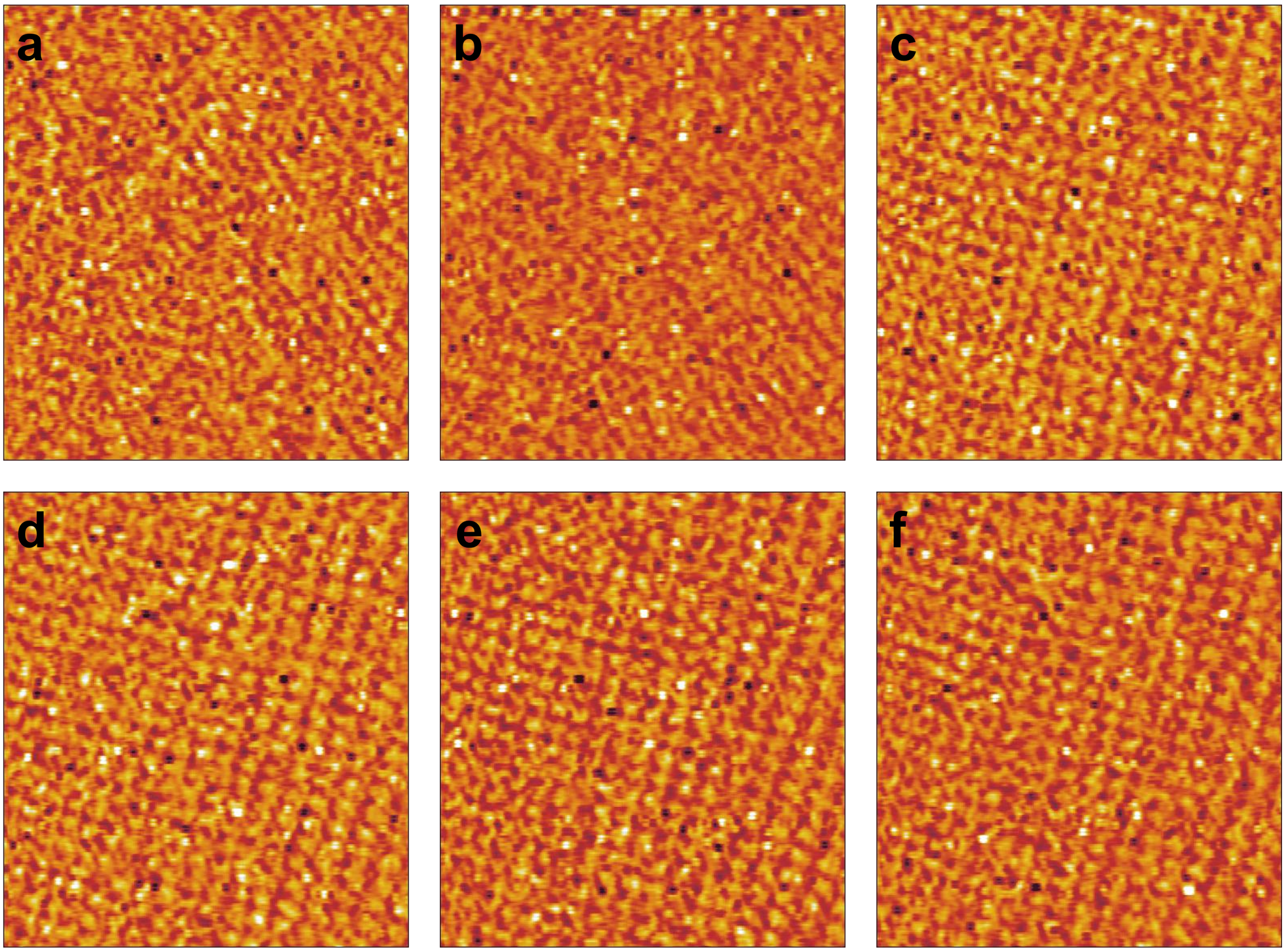

Figure S11. Sequence of subsequent recorded video-STM images at time intervals of 0.1 s, recorded on Ag(100) in 1 mM KBr + 1mM $NaClO_4$ at -0.54 V (4 °C), i.e., the potential of the order-disorder phase transition in the Br adlayer. Fluctuations between a local (1×1) and c(2×2) lattice can be observed, as expected near the Ising phase transition. No clear domain boundaries can be observed, which we attribute to the high dynamics within the halide adlayer, similar as observed near c(2×2) domain boundaries in a very similar adsorbate system[16].

3. Details on the density functional calculations

3.1. Computational details

Periodic density-functional total-energy calculations with plane-waves basis-set have been carried through using `PWscf` and `PWneb` from the code package QUANTUM ESPRESSO (version 6.6)[17,18]. The Ag(100) surface has been modeled by a periodically repeated slab geometry. A p(6×6) surface unit cell has been employed and the slabs consisted of 6, 8, ... up to 16 Ag layers. The slabs were asymmetric, i.e. the adsorbates were located on only one side, while the two bottommost layers have always been kept fixed at Ag bulk positions. A dipole correction has been applied with a vacuum region thickness of approximately 13 Å.

The exchange-correlation functional has been approximated with the generalized gradient approximation (GGA) by Perdew, Burke and Ernzerhof (PBE)[19]. An energy cutoff of 30 Ry $\approx$ 408 eV has been applied for expanding the electronic wavefunctions in a plane-wave basis set. Ionic potentials were modeled by PAW pseudopotentials[20] from the PSLibrary 0.3.1 created by Dal Corso[21]. A $3 \times 3 \times 1$ mesh of special **k**-points according to Monkhorst and Pack[22] has been used for Brillouin zone integration, resulting in 5 **k**-points in the irreducible wedge of the Brillouin zone. No symmetry apart from time reversal has been assumed. Electronic energies have been considered converged when energy changes between self consistency iterations were below 10^{-6} Ry $\approx 1.36 \cdot 10^{-5}$ eV. Atomic geometries have been relaxed as long as forces between atoms exceeded 10^{-4} Ry/bohr ≈ 0.0026 eV/Å.

Minimum energy paths and transition states of the S_{ad} diffusion have been obtained by employing the climbing-image nudged elastic band (CI-NEB) method[23,24] as implemented in `PWneb` from QUANTUM ESPRESSO. On that account adatom configurations corresponding to local minima of the potential energy surface of the system were concatenated by paths with three interpolated intermediate configurations, *i.e.* the NEB paths consisted of five configurations (images) in total. Image interpolation for the initial NEB paths was obtained with the image

dependent pair potential (IDPP) method [25] as implemented in the Atomic Simulation Environment (ASE) [26] for Python. Optimizations of NEB paths were performed until the magnitude of the force orthogonal to the path was less than 0.05 eV/Å. The NEB calculations have been carried out with six Ag layers. To obtain S^{TS}–Br interaction energies for more than six Ag layers, two or more layers with Ag bulk atomic coordinates were added to the bottom of the slab of the transition state configuration as well as of the corresponding configurations with the single S or Br on the surface and of the clean surface. Then these configurations with added layers have been relaxed only electronically. Tests for NEB runs with eight geometrically relaxed Ag layers showed differences of only a few meV in the S activation energy compared to configurations obtained with six layers NEB runs which were electronically relaxed after adding two layers in the initial and TS configurations. This procedure was necessary because NEB calculations with full ionic relaxation with up to 16 Ag layers and five images were not computationally feasible in a reasonable amount of time and in view of very large memory requirements.
For the calculations presented here we used our calculated Ag bulk lattice constant of 7.856 bohr ≈ 4.157 Å which is in agreement with results from the literature with the same XC-functional and pseudopotential, e.g. [21].

3.2. Br–Br and S–Br interaction energy on Ag(100)

3.2.1. Definition of the interaction energy

The interaction energy ΔE_{int} between an X adatom (X = Br or X = S) and a configuration $\mathcal{C}_{\text{Br}}$ of Br adatoms on Ag(100) has been calculated as

$$\Delta E_{\text{int}} = E(\text{X}, \mathcal{C}_{\text{Br}}/\text{Ag}(100)) + E(\text{Ag}(100)) - [E(\mathcal{C}_{\text{Br}}/\text{Ag}(100)) + E(\text{X}/\text{Ag}(100))]\,. \quad (1)$$

$E(\text{X}, \mathcal{C}_{\text{Br}}/\text{Ag}(100))$ denotes the DFT total energy of the Ag(100) slab with an adsorbed X atom and one, two or three Br adatoms adsorbed in configuration $\mathcal{C}_{\text{Br}}$, while $E(\text{Ag}(100))$ stands for the total energy of the clean Ag(100) slab. $E(\mathcal{C}_{\text{Br}}/\text{Ag}(100))$ is the total energy of only the Br adatoms in configuration $\mathcal{C}_{\text{Br}}$ adsorbed on the surface and $E(\text{X}/\text{Ag}(100))$ is the total energy of only the X atom adsorbed on the slab. With that definition $\Delta E_{\text{int}} > 0$ refers to a decrease of the absolute value of the X adsorption energy due to the coadsorbed Br adatoms as compared to X adsorption on clean Ag(100).

3.2.2. Adatom configurations

For the MC simulations presented in this paper the interaction energies of two adsorbate atoms, S–Br and Br–Br, on Ag(100) have been calculated. Configurations contain one Br and one S adatom (or two Br adatoms, respectively) in a p(6×6)-Ag(100) surface unit cell. The eight symmetrically inequivalent S-Br and Br-Br configurations with a lateral adatom-adatom separation larger than the nearest-neighbour (NN) distance are shown in Figs. S12b to S12i. The interaction energies referring to these adsorbate configurations are shown in Fig. S16.

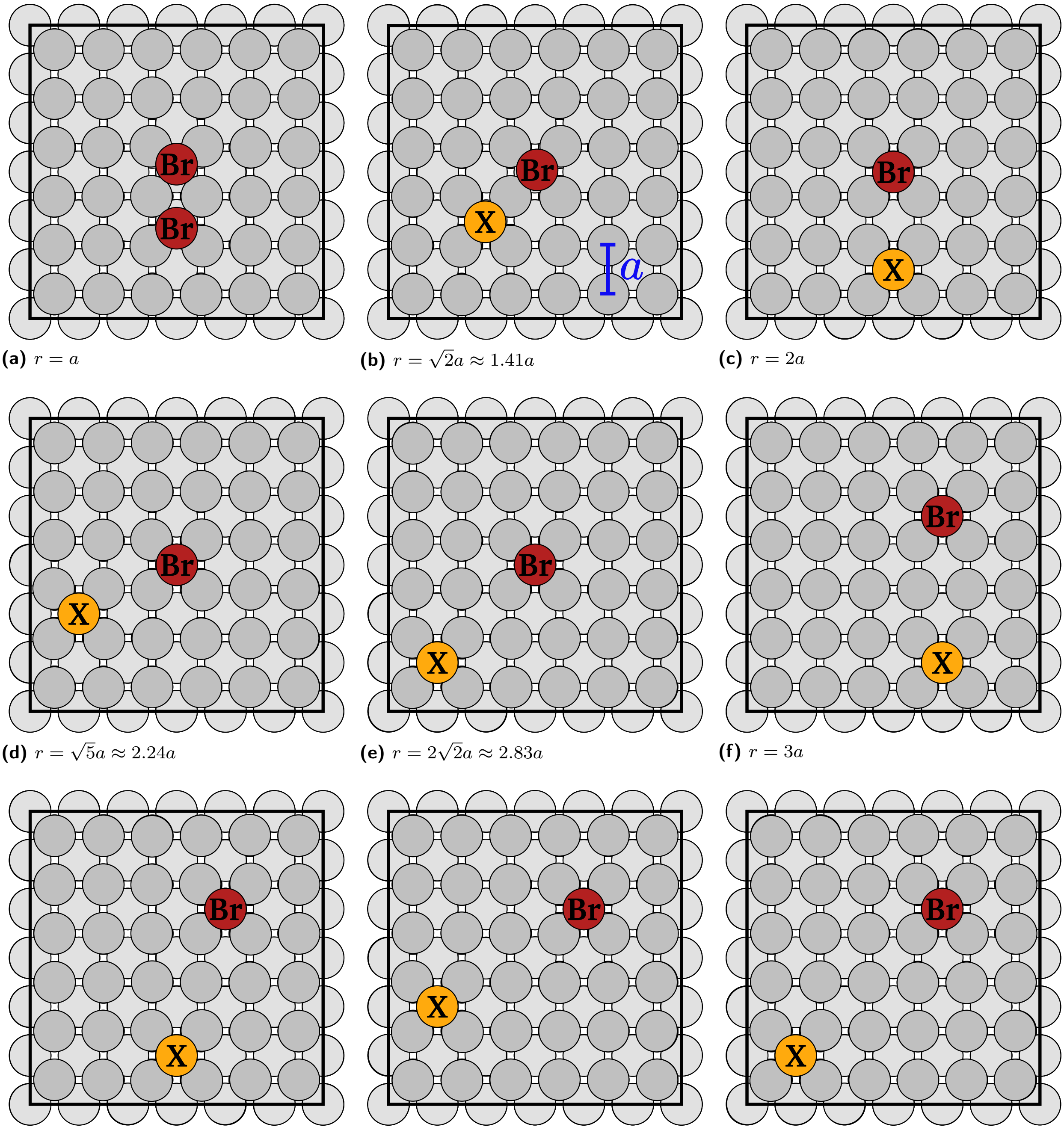

(a) $r = a$ **(b)** $r = \sqrt{2}a \approx 1.41a$ **(c)** $r = 2a$

(d) $r = \sqrt{5}a \approx 2.24a$ **(e)** $r = 2\sqrt{2}a \approx 2.83a$ **(f)** $r = 3a$

(g) $r = \sqrt{10}a \approx 3.16a$ **(h)** $r = \sqrt{13}a \approx 3.61a$ **(i)** $r = 3\sqrt{2}a \approx 4.24a$

Figure S12. Relaxed X–Br configurations (X = Br or X = S) used to calculate Br–Br and S–Br interaction energies. Both adatoms are in adsorption position (hollows). Below each configuration the lateral distance r between the centers of the hollow sites containing the adatoms is noted, i.e. r does not account for adatom relaxations; a is the lattice constant of the Ag(100) p(1×1)-surface. (a) shows two Br adatoms in NN distance. (b)-(i) show all eight symmetrically inequivalent X–Br configurations in the p(6×6)-Ag(100) surface cell (black frames) with r larger than the NN distance. Ag atoms of the topmost and second layer are drawn as dark and light gray circles, respectively.

The Br–Br interaction energy of NN Br adatoms (Fig. S12a) can be found in Table S2 for various number of Ag substrate layers. For one S and one Br adatom in NN configuration the atomic relaxation of the system resulted in a displacement of the Br adatom into the neighboring hollow position located straight away from the S position (i.e. the configuration in Fig. S12c). For this reason the S–Br interaction energy for the S–Br NN distance cannot be determined directly. To derive an estimate for the NN S–Br interaction the interaction energy of the configuration with two Br adatoms shown in Fig. S13a has been calculated; the presence of the second Br adatom prevents the other Br adatom from leaving its NN hollow position and relaxing into a neighboring hollow site of the substrate. Following the ansatz of employing a superposition of S–Br (and Br–Br) pair interactions

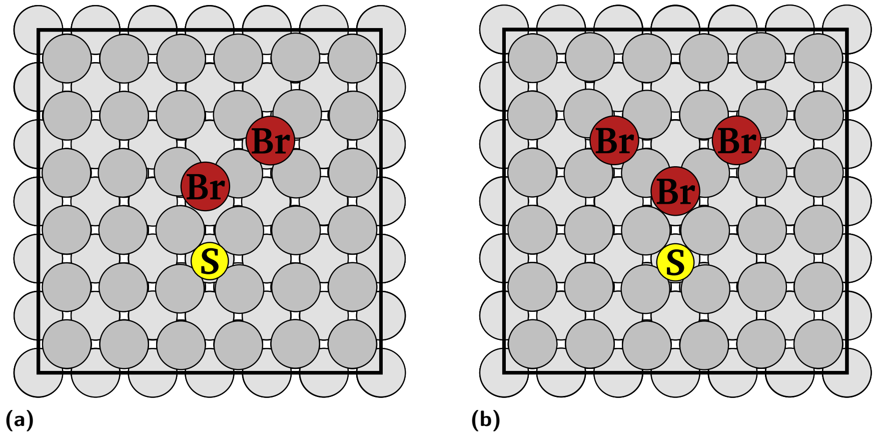

Figure S13. Stabilization of S–Br NN configuration by one or two additional Br adatoms. Relaxed S–Br configurations are shown, which are calculated to obtain estimates for the S–Br interaction energy for S and Br in NN configuration.

in our MC simulations, an estimate for the interaction of the S adatom with one Br adatom in NN distance has then be obtained by subtracting the S–Br interaction energy of the S–Br configuration in Fig. S12d from the S–Br interaction energy of the S–Br configuration with two Br adatoms in Fig. S13a. The results for this estimate for different numbers of Ag layers are also noted in Table S2.
To obtain, for a comparison, yet another estimate for the S–Br NN interaction, the S–Br configuration in Fig. S13b with two stabilizing Br adatoms (i.e. three Br_{ad} in total) has been calculated. Analogously, the S–Br NN interaction has been attained by subtracting two times (due to symmetry) the interaction energy of the configuration shown in Fig. S12d from the one of the configuration in S13b. The results for this estimate for different numbers of Ag layers are noted in Table S2 as well.
The interaction energy of S and Br adsorbed at NN sites is ≈ 240 meV in case of a single additional stabilizing Br adatom and ≈ 320 meV with two additional stabilizing Br adatoms. Due to the large repulsive value of this interaction energy this difference does not significantly affect the MC results for the average S_{ad} hollow-bridge-hollow hopping rate discussed below.

Table S2. DFT Br–Br interaction energy $\Delta E^{\text{Br-Br}}_{\text{int,NN}}$ on Ag(100) for Br and Br in NN distance ($r = a$), estimate $\Delta E^{\text{S-Br}}_{\text{int,NN}}$ for the DFT S–Br interaction energy for S and Br in NN distance (obtained as explained in the text) and estimates $\Delta E^{\text{S}^{\text{TS}}\text{-Br}}_{\text{int,1}}$ and $\Delta E^{\text{S}^{\text{TS}}\text{-Br}}_{\text{int,2}}$ for the DFT S^{TS}-Br interaction energy for S^{TS} and Br in the two smallest lateral S^{TS}-Br distances $r = \sqrt{1.25} \approx 1.12a$ and $r = 1.5a$ respectively (obtained as explained in the text) vs. number of Ag layers used in the DFT calculations. Energy values in meV.

	no. of Ag layers	10	12	14	16
**Br–Br**	$\Delta E^{\text{Br-Br}}_{\text{int,NN}}$	150	153	153	151
**S–Br**	$\Delta E^{\text{S-Br}}_{\text{int,NN}}$ (1 additional Br_{ad})	238	244	238	237
	$\Delta E^{\text{S-Br}}_{\text{int,NN}}$ (2 additional Br_{ad})	316	324	314	313
**S^{TS}–Br**	$\Delta E^{\text{S}^{\text{TS}}\text{-Br}}_{\text{int,1}}$ (1 additional Br_{ad})	210	211	205	207
	$\Delta E^{\text{S}^{\text{TS}}\text{-Br}}_{\text{int,2}}$ (1 additional Br_{ad})	16	13	13	12
	$\Delta E^{\text{S}^{\text{TS}}\text{-Br}}_{\text{int,1}}$ (2 additional Br_{ad})	288	290	281	284
	$\Delta E^{\text{S}^{\text{TS}}\text{-Br}}_{\text{int,2}}$ (2 additional Br_{ad})	20	15	12	12

Fig. S14 shows the S^{TS}–Br configurations used to calculate the S^{TS}–Br interaction energies between a Br adatom and the S adatom in the transition state (TS) of a hollow-bridge-hollow diffusion hop.

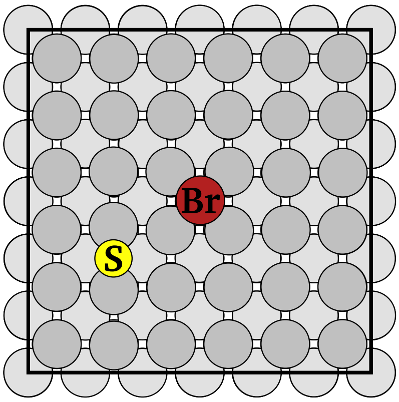

(a) $r = \sqrt{3.25}a \approx 1.80a$

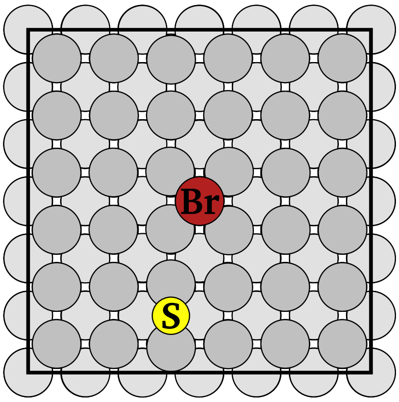

(b) $r = \sqrt{4.25}a \approx 2.06a$

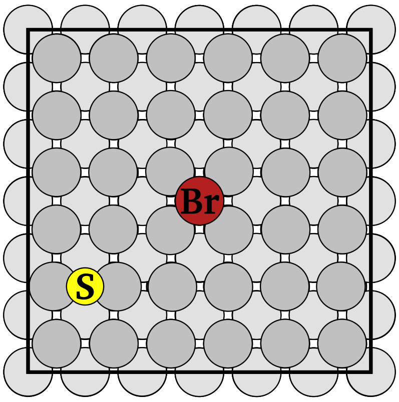

(c) $r = \sqrt{6.25}a = 2.5a$

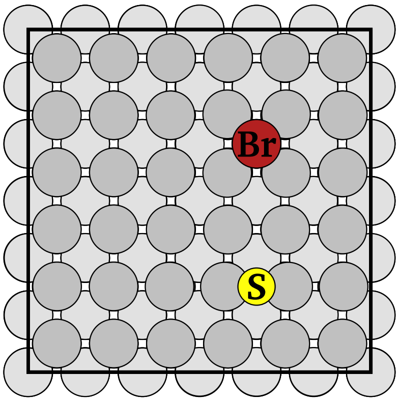

(d) $r = 2.5a$

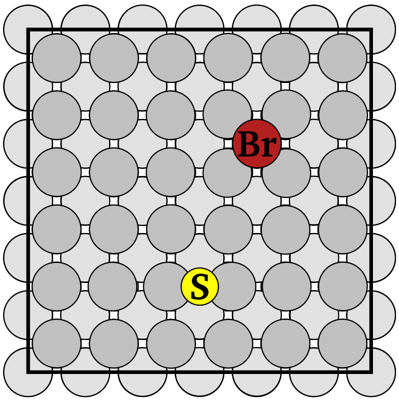

(e) $r = \sqrt{7.25}a \approx 2.69a$

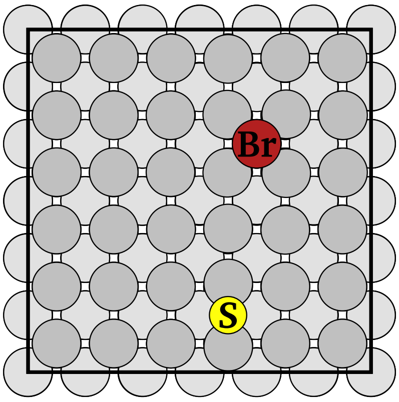

(f) $r = \sqrt{9.25}a \approx 3.04a$

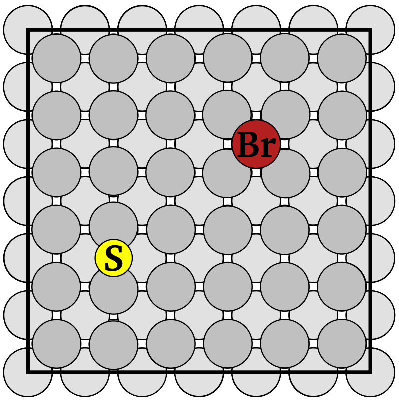

(g) $r = \sqrt{10.25}a \approx 3.20a$

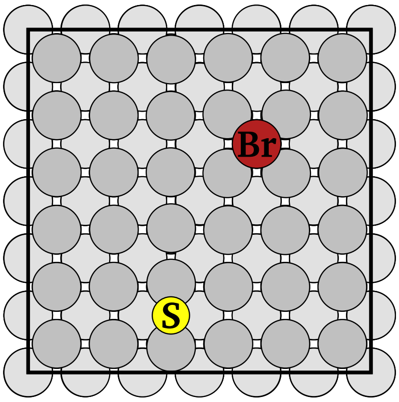

(h) $r = \sqrt{11.25}a \approx 3.25a$

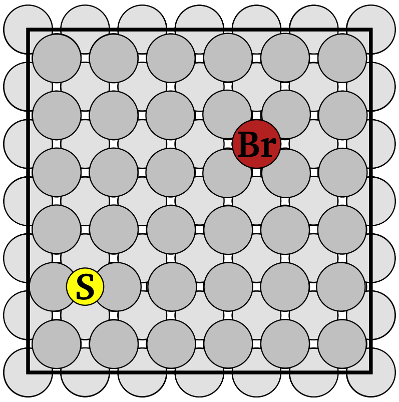

(i) $r = \sqrt{15.25}a \approx 3.91a$

Figure S14. S^{TS}–Br configurations used to calculate the S^{TS}–Br interaction energies for the MC simulations. The Br adatom is in its adsorption position and the S adatom is located in the transition state (TS) of a hollow-bridge-hollow hop (calculated with NEB in the presence of Br_{ad}). Below each configuration the lateral distance r between the centers of the respective unrelaxed bridge and hollow sites is noted. (a)-(i) show all possible nine symmetrically inequivalent S^{TS}–Br configurations with $r > 1.5a$ in the p(6×6)-Ag(100) surface cell.

Figs. S14a to S14i show the nine symmetrically inequivalent S^{TS}–Br configurations in the p(6 × 6)-Ag(100) surface unit cell with lateral adatom-adatom separations $r > 1.5a$. The corresponding S^{TS}–Br interaction energies are displayed in Fig. S16. Two more symmetrically inequivalent S^{TS}–Br configurations exist, with $r \leq 1.5a$ (compare Fig. S15). These correspond to hollow-bridge-hollow diffusion paths of the S adatom for which in either the initial or the final configuration of the diffusion path S and Br would end up in an unstable NN configuration. These paths can not be calculated due to the geometric instability mentioned above, *i.e.* that the Br adatom relaxes out of its hollow site, when it initially is NN to the S adatom. Similar to the procedure applied above to derive the S–Br NN-interaction energy, in order to obtain estimates for the S^{TS}–Br interaction for the two configurations with $r \leq 1.5a$, the S^{TS}–Br interaction energies of the two configurations with two Br adatoms shown in Figs. S15a left and S15b left have been calculated and from each of these interaction energies the S^{TS}–Br interaction energies of the configurations in Figs. S14b and S14e have been subtracted. The resulting interaction energy estimates for lateral S^{TS}–Br distances $r = \sqrt{1.25} \approx 1.12a$ and $r = 1.5a$ are also noted in table S2.

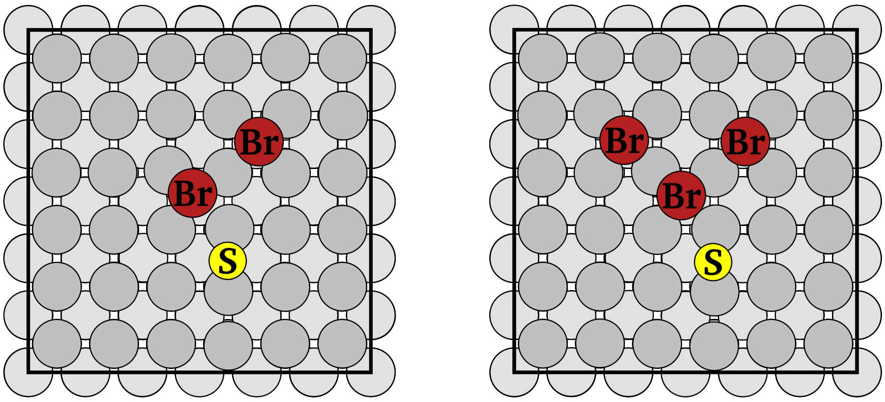

(a) $r = \sqrt{1.25}a \approx 1.12a$

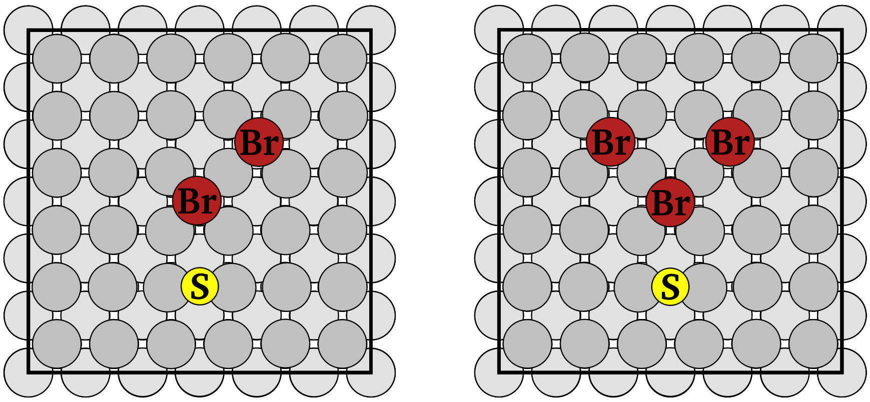

(b) $r = 1.5a$

Figure S15. S^{TS}–Br configurations with one (top row) or two (bottom row) additional Br adatoms used to stabilize the diffusion paths for S starting or ending in S–Br NN configuration (calculated with NEB in the presence of Br_{ad}). Below the configurations the lateral distance r between the bridge site of the S_{ad} before relaxation and the hollow site of the Br_{ad} closest to the S_{ad} is noted.

Again, an analogous approach has been taken to obtain corresponding estimates for the S^{TS}–Br interaction for the two configurations with $r \leq 1.5a$ based on two additional stabilizing Br adatoms. For that, the configurations in Figs. S15a right and S15b right have been calculated and from the interaction energy of the former structure the interaction energies of the configurations in Figs. S14b and S14c have been subtracted, while two times (due to symmetry) the interaction energy of the configuration in Fig. S14e has been subtracted from the latter. Once more the resulting estimates for various number of Ag layers can be found in Table S2. With a single additional Br_{ad} for stabilization, the S^{TS}-Br interaction energy amounts to ≈ 207 meV or ≈ 284 meV (for sideway moves) and ≈ 12 meV (when S hops straight away from the Br), respectively. With two stabilizing Br_{ad}, interaction energies amount to ≈ 288 meV (for sideway moves) and ≈ 15 meV (when S hops straight away from the Br), respectively. We again verified that this variation does not significantly affect the average S_{ad} hopping rate discussed below.

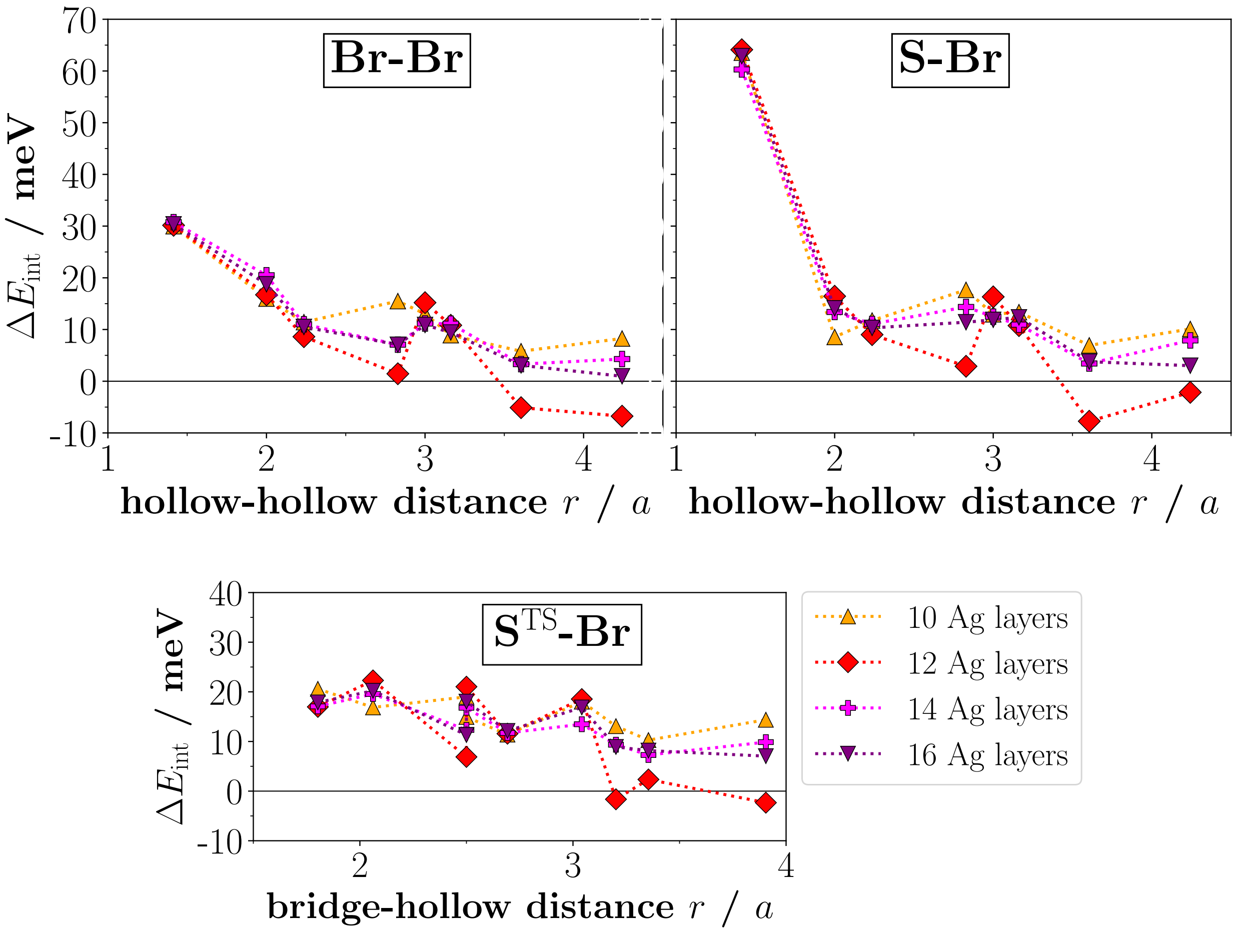

Figure S16. DFT interaction energy of two Br or one Br and one S adsorbate atom in the p(6×6)-Ag(100) surface unit cell vs. the lateral separation r. The corresponding Br-Br, S-Br and S^{TS}-Br configurations are visualized in Figs. S12 and S14. a is the lattice constant of the p(1×1) Ag(100) surface. Dotted lines are guide to the eye.

3.2.3. Accuracy of the DFT interaction energy

Detailed convergence tests, not shown here, with respect to plane wave cutoff energy, number of **k**-points and thickness of vacuum for the bridge-hollow total energy difference of an S_{ad} or Br_{ad} in a p(2×2) cell suggest an accuracy of DFT total-energy differences of ±8 meV. This estimate does not account for the inaccuracy due to the approximate XC-functional and slab thickness. The effect of slab thickness on the Br–Br, S–Br and S^{TS}–Br interaction energies can be read from Tab. S2 and Fig. S16. Starting from 14 Ag layers the variation of DFT interaction energies due to slab thickness is below 5 meV. This yields a total accuracy of the Br-Br and S-Br interaction energies of ±13 meV for results obtained with at least 14 Ag layers. In case of the S^{TS}–Br interaction an increase of the number of NEB images from 5 to 7 showed a variation of 2 meV of the S energy barrier in presence of a Br co-adsorbate, which results in a total accuracy of ±15 meV of the S^{TS}–Br interaction obtained with at least 14 Ag layers.

4. Details on the lattice gas model

4.1. Derivation of the average S_{ad} hollow-bridge-hollow hopping rate

Modelling the lattice gas starts from an $L \times L$ square unit cell of adsorption sites (later on $L = 6$); each site representing a four-fold hollow position on the Ag(100) substrate surface. Similar to ref.[27] we assign an energy to each configuration $\mathcal{C}_{\mathrm{Br}}$ of Br adatoms by means of the Hamiltonian

$$H^{\mathcal{C}_{\mathrm{Br}}} = N^{\mathrm{Br}} E^{\mathrm{Br}}_{\mathrm{ads}} + \frac{1}{2} \sum_{i \neq j} \Phi^{\mathrm{Br\text{-}Br}}_{ij} \sigma_i \sigma_j \ . \tag{2}$$

σ_i equals 1 if the site i is occupied with Br_ad and 0 otherwise. $\Phi_{ij}^\text{Br-Br}$ is the lateral interaction energy of two Br adatoms located on sites i and j, while E_ads^Br denotes the adsorption energy of a single Br adatom in the $L \times L$ surface unit cell. $\Phi_{ij}^\text{Br-Br} > 0$ implies repulsive interaction and $E_\text{ads}^\text{Br} < 0$ favors adsorption. $N^\text{Br} = \sum_{i=1}^{L^2} \sigma_i$ is the number of Br_ad adsorbed within the surface unit cell. Now consider an additional single S adatom adsorbed at site i_S. There must not be any Br_ad at this site, i.e. $\sigma_{i_\text{S}} = 0$. The Hamiltonian H of the S/Br/Ag(100) lattice gas is

$$H = H^{\mathcal{C}_\text{Br}} + E_\text{ads}^\text{S} + \sum_{j=1,\ j\neq i_S}^{L^2} \Phi_{i_\text{S}j}^\text{S-Br} \sigma_j \ . \tag{3}$$

E_ads^S denotes the S adsorption energy and $\Phi_{i_\text{S}j}^\text{S-Br}$ the lateral S_ad-Br_ad interaction energy for an S adatom on site i_S and a Br adatom on site j.

For the S/Br/Ag(100) lattice gas model we assume that diffusion of the sulfur adatom takes place by a hop of S_ad to a neighboring empty hollow site via a bridge site of the substrate lattice. S diffusion hops via an Ag vacancy as discussed in the paper and further down below are not included in the lattice gas model. In our model, during the crossing of the transition state by S_ad the configuration of Br adatoms does not change. The DFT activation energy barriers for the hollow-bridge-hollow diffusion path of Br or S on a clean p(6×6)-Ag(100) surface amount to $E_\text{A, p6×6}^\text{Br,bridge} \approx 135$ meV and $E_\text{A, p6×6}^\text{S,bridge} \approx 745$ meV, in good agreement to [28] and [29]. Assuming attempt frequencies for the hops of similar order of magnitude, the Br_ad hopping rate will be many orders of magnitude larger than the S_ad hopping rate. Therefore, as long as not locked to a $c(2\times 2)$ configuration of the Br adlattice, the S adatom is sampling a thermodynamic equilibrium ensemble of Br_ad configurations between two hops. Transition state theory [30] is applied for the hopping rate of S_ad at a fixed Br_ad configuration,

$$\nu_{\mathcal{C}_\text{Br}}^\text{S} = \nu_0^\text{S} \cdot \exp\left(-\frac{1}{k_\text{B}T} E_\text{A}^{\text{S},\mathcal{C}_\text{Br}}\right) \ . \tag{4}$$

k_B is Boltzmann's constant, T the temperature, ν_0^S the attempt frequency for an S_ad hop on the clean surface (any effect of Br co-adsorbates on the prefactor is neglected) and $E_\text{A}^{\text{S},\mathcal{C}_\text{Br}}$ the activation energy barrier for an S_ad hop with the Br spectator adatoms in the configuration $\mathcal{C}_\text{Br}$. The activation energy is given by the energy difference of the system with S in the transition state (close to a bridge site) and at the adsorption site (*i.e.* at a hollow site of the Ag(100) substrate). The energy of the system with a single S adsorbate located at such a TS site is described by

$$H^\text{TS} = H^{\mathcal{C}_\text{Br}} + E_\text{ads}^\text{S,TS} + \sum_{j=1}^{L^2} \Phi_{i_\text{S}\to i'_\text{S},j}^\text{S-Br} \sigma_j \ . \tag{5}$$

i_S and i'_S are the lattice sites between which the S_ad hops. For an S_ad hop to be possible, both the initial and the final site of the associated S hop have to be unoccupied by Br, *i.e.* $\sigma_{i_\text{S}} = \sigma_{i'_\text{S}} = 0$. $E_\text{ads}^\text{S,TS}$ is the adsorption energy of the S adatom in the transition state on an otherwise clean Ag(100) surface and $\Phi_{i_\text{S}\to i'_\text{S},j}^\text{S-Br}$ denotes the lateral interaction energy of the S adsorbate in the TS and a Br adatom in site j. The activation energy in Eq. (4) is given by the energy difference $E_\text{A}^{\text{S},\mathcal{C}_\text{Br}} = H^\text{TS} - H$, which yields

$$E_\text{A}^{\text{S},\mathcal{C}_\text{Br}} = E_\text{A}^\text{S,clean} + \sum_{j=1}^{L^2} \left(\Phi_{i_\text{S}\to i'_\text{S},j}^\text{S-Br} - \Phi_{i_\text{S}j}^\text{S-Br}\right) \sigma_j, \tag{6}$$

where $E_\text{A}^\text{S,clean} := E_\text{ads}^\text{S,TS} - E_\text{ads}^\text{S}$ describes the activation energy for a hop of S_ad on an otherwise clean Ag(100) surface.

Following Eqs. (4) and (6) the rate of the S adatom hopping away from its adsorption site i_S to a specific neighboring site i'_S, interacting with a fixed configuration $\mathcal{C}_{\text{Br}^r}$ of Br adsorbates, is

$$\nu_{\mathcal{C}_\text{Br}}^\text{S} = \left(1 - \sigma_{i'_\text{S}}\right) \cdot \nu_0^\text{S} \cdot \exp\left(-\frac{1}{k_\text{B}T}\left[E_\text{A}^\text{S,clean} + \sum_{j=1}^{L^2}\left(\Phi_{i_\text{S}\to i'_\text{S},j}^\text{S-Br} - \Phi_{i_\text{S}j}^\text{S-Br}\right)\sigma_j\right]\right) \ . \tag{7}$$

The prefactor $\left(1 - \sigma_{i'_\text{S}}\right)$ reflects the constraint, that the target site i'_S of the S hop has to be unoccupied by Br. Elsewise the hopping rate vanishes due to site blocking. With the S hopping rate on the clean Ag(100) surface $\nu_\text{clean}^\text{S}$ this rate can be written in the form

$$\nu_{\mathcal{C}_\text{Br}}^\text{S} = \left(1 - \sigma_{i'_\text{S}}\right) \cdot \frac{\nu_\text{clean}^\text{S}}{4} \cdot \exp\left(-\frac{1}{k_\text{B}T}\sum_{j=1}^{L^2}\left(\Phi_{i_\text{S}\to i'_\text{S},j}^\text{S-Br} - \Phi_{i_\text{S}j}^\text{S-Br}\right)\sigma_j\right) . \tag{8}$$

The factor 1/4 accounts for the four directions allowed for S adatom hopping on the Ag(100) substrate surface. Considering the different time scales it takes the adatoms to transverse the transition geometry and the largely different inverse hopping rates of Br and S on Ag(100), as long as the S_{ad} is not locked to a c(2×2) sublattice of the Br adatoms, the hopping rate $\bar{\nu}^{S}$ of the S adatom away from its adsorption site in any single direction can be derived from the grand canonical average of all $\nu^{S}_{\mathcal{C}_{Br}}$ at fixed configurations of Br adsorption sites with respect to the Br_{ad} configurations:

$$\bar{\nu}^{S} = \frac{\sum\limits_{\sigma_1 \in \{0,1\}} \cdots \sum\limits_{\sigma_{L^2} \in \{0,1\}} (1-\sigma_{i_S})\left(1-\sigma_{i'_S}\right) \frac{\nu^{S}_{clean}}{4} e^{-\beta \sum_{j=1}^{L^2} \left(\Phi^{S\text{-}Br}_{i_S \to i'_S, j} - \Phi^{S\text{-}Br}_{i_S j}\right)\sigma_j} e^{-\beta\left(H - N^{Br}\mu^{Br}\right)}}{\sum\limits_{\sigma_1 \in \{0,1\}} \cdots \sum\limits_{\sigma_{L^2} \in \{0,1\}} (1-\sigma_{i_S})\, e^{-\beta\left(H - N^{Br}\mu^{Br}\right)}}. \tag{9}$$

μ^{Br} denotes the chemical potential of the Br adatoms, $\beta := 1/k_B T$. The summation now runs over all Br adatom configurations. The factor $1-\sigma_{i_S}$ excludes configurations from the summation where site i_S would be falsely occupied by a Br adatom. All constraints on the allowed Br adatom configurations are included explicitly by the factors $1-\sigma_{i_S}$ and $1-\sigma_{i'_S}$ in the numerator as well as in the denominator.
Now consider the expectation value of a physical quantity $X^{\mathcal{C}_{Br}}$, which depends on the Br configuration $\mathcal{C}_{Br}$, with respect to the grand canonical ensemble of all possible Br adatom configurations (without any S_{ad}). With the total energy $H^{\mathcal{C}_{Br}}$ of the Br/Ag(100) subsystem (Eq. (2)) this can be written as

$$\left\langle X^{\mathcal{C}_{Br}} \right\rangle := \frac{\sum\limits_{\sigma_1 \in \{0,1\}} \cdots \sum\limits_{\sigma_{L^2} \in \{0,1\}} X^{\mathcal{C}_{Br}} e^{-\beta\left(H^{\mathcal{C}_{Br}} - N^{Br}\mu^{Br}\right)}}{\sum\limits_{\sigma_1 \in \{0,1\}} \cdots \sum\limits_{\sigma_{L^2} \in \{0,1\}} e^{-\beta\left(H^{\mathcal{C}_{Br}} - N^{Br}\mu^{Br}\right)}}. \tag{10}$$

Dividing numerator and denominator in Eq. (9) by the grand canonical partition sum of the Br adsorbate configurations and using Eq. (3) thus yields

$$\bar{\nu}^{S} = \frac{\nu^{S}_{clean}}{4} \frac{\left\langle (1-\sigma_{i_S})\left(1-\sigma_{i'_S}\right) e^{-\beta \sum_{j=1}^{L^2}\left(\Phi^{S\text{-}Br}_{i_S \to i'_S, j} - \Phi^{S\text{-}Br}_{i_S j}\right)\sigma_j - \beta\left(E^{S}_{ads} + \sum_{j=1}^{L^2} \Phi^{S\text{-}Br}_{i_S j}\sigma_j\right)} \right\rangle}{\left\langle (1-\sigma_{i_S})\, e^{-\beta\left(E^{S}_{ads} + \sum_{j=1}^{L^2} \Phi^{S\text{-}Br}_{i_S j}\sigma_j\right)} \right\rangle}. \tag{11}$$

By division of the constant quantity $\exp\left(-\beta E^{S}_{ads}\right)$ from the numerator and the denominator one finally arrives at

$$\bar{\nu}^{S} = \frac{\nu^{S}_{clean}}{4} \frac{\left\langle (1-\sigma_{i_S})(1-\sigma_{i'_S}) \exp\left(-\frac{1}{k_B T} \sum_{j=1}^{L^2} \Phi^{S\text{-}Br}_{i_S \to i'_S, j}\sigma_j\right) \right\rangle}{\left\langle (1-\sigma_{i_S}) \exp\left(-\frac{1}{k_B T} \sum_{j=1}^{L^2} \Phi^{S\text{-}Br}_{i_S, j}\sigma_j\right) \right\rangle}, \tag{12}$$

which yields the quotient $h := \bar{\nu}^{S} / \left(\frac{1}{4}\nu^{S}_{clean}\right)$. Given the sets of interaction energies Φ for the Br-Br, S-Br and S^{TS}-Br interaction, the quantity h can readily be computed by equilibrium grand canonical Monte Carlo simulations at some given Br chemical potential μ_{Br}. On the clean Ag(100) surface, $\bar{\nu}^{S} = \frac{1}{4}\nu^{S}_{clean}$ and $h = 1$.

4.2. Details on the Monte Carlo simulations

The Monte Carlo simulations have been carried out in the grand canonical ensemble by switching the Br occupation of lattice sites. The size of the simulation cell was set to $L = 6$ in order to directly use the DFT interaction energy parameters without need for extrapolation to a larger cell size. The value of $\mu^{Br} - E^{Br}_{ads}$ (Eqs. (2) and (10)) has been preset for each single MC simulation. MC expectation values of the form of Eq. (10) have been calculated. Several MC simulations have been performed in sequence for $\mu^{Br} - E^{Br}_{ads}$ increased from -200 meV to 500 meV in steps of 10 meV. The sequence was initialized with a Br coverage of $\theta = 0$ and $\mu^{Br} - E^{Br}_{ads} = -200$ meV and each remaining MC simulation of the sequence started with the Br configuration of the preceding simulation. Each simulation comprised $8 \cdot 10^6$ lattice sweeps for equilibration; one lattice sweep consists of a loop over all $L^2 = 36$ lattice sites and attempting to switch the Br occupation of each site exactly once according to the Metropolis algorithm[31]. After equilibration, $4 \cdot 10^6$ lattice sweeps are used for accumulating MC averages. The respective quantities have been calculated after every single sweep. The number of sweeps has been chosen large enough, so that an increase does not affect the result for $h(\theta)$. A large number of sweeps is required in order to obtain accurate MC averages for *e.g* the denominator in Eqs. (12) and (13) in case of Br coverage $\theta \to 1/2$. This is due to the rare occurrence of particular Br_{ad} configurations at large intervals in the MC trajectory that produce

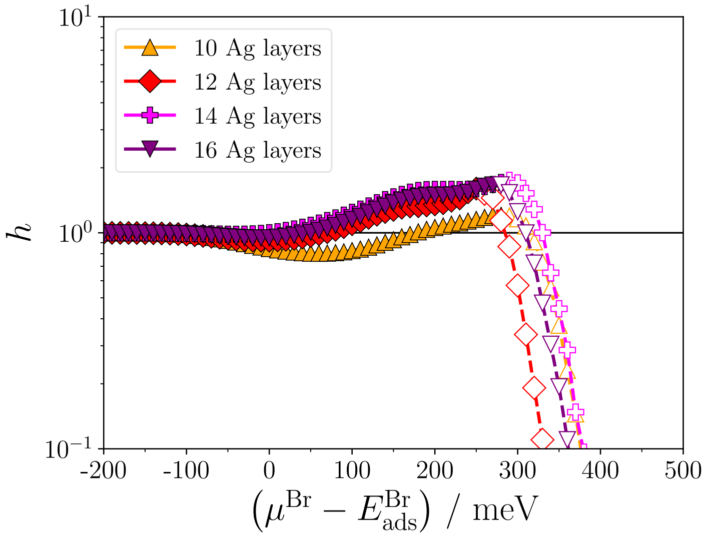

Figure S17. Quotient h of S hopping rates (Eq. (13)) at room temperature in the presence of Br co-adsorbates vs. Br chemical potential. Open symbols indicate the range of Br chemical potential where for higher $\mathrm{Br_{ad}}$ coverage more complex S diffusion paths neglected in the lattice gas approach may become relevant.

large individual contributions to the respective expectation values. The random number generator `Ran` from[32] has been used for the Metropolis procedure. For the calculation of the scaling factor h (Eq. (12)) the final $\mathrm{S_{ad}}$ position $i'_{\rm S}$ has always been chosen as the NN position to the right of the initial $\mathrm{S_{ad}}$ position $i_{\rm S}$. Furthermore the actual calculation has been carried out by letting the $\mathrm{S_{ad}}$ position $i_{\rm S}$ run over all lattice sites except the sites on the far right edge of the cell and averaging over these before taking the MC averages $\langle . \rangle$ in the numerator and denominator, i.e.

$$h = \frac{\left\langle {\sum}'^{L^2-L}_{i_{\rm S}=1} \left[(1-\sigma_{i_{\rm S}})(1-\sigma_{i'_{\rm S}}) \exp\left(-\frac{1}{k_{\rm B}T}\sum_{j=1}^{L^2} \Phi^{\text{S-Br}}_{i_{\rm S}\to i'_{\rm S},j}\sigma_j\right)\right]\right\rangle}{\left\langle {\sum}'^{L^2-L}_{i_{\rm S}=1} \left[(1-\sigma_{i_{\rm S}}) \exp\left(-\frac{1}{k_{\rm B}T}\sum_{j=1}^{L^2} \Phi^{\text{S-Br}}_{i_{\rm S},j}\sigma_j\right)\right]\right\rangle}, \tag{13}$$

where ${\sum}'^{L^2-L}_{i_{\rm S}=1}$ stands for a summation over $L^2 - L$ sites, leaving out the L sites on the right edge of the $L \times L$ cell. This site averaging procedure avoids that $i_{\rm S}$ coincidentally lies in an outlasting c(2×2) patch of the Br adlayer leading always to $h = 0$ because of the factors $(1 - \sigma_{i_{\rm S}})(1 - \sigma_{i'_{\rm S}})$ in the numerator, which could be the case especially for higher but not everywhere saturated Br coverage. The MC results have been calculated with a lattice size of $L = 6$ with Br-Br, S-Br and $\mathrm{S^{TS}}$-Br interaction energies obtained from DFT in a p(6×6) surface cell, i.e. the same size as in the MC simulations. In the MC simulations no explicit periodic boundary conditions had to be applied to the Hamiltonian, since the DFT interaction energies already ensure the correct 6×6 translational symmetry due to the periodic boundary conditions in the DFT calculations.

In Fig. S17 the quotient h of the MC average of the $\mathrm{S_{ad}}$ hollow-bridge-hollow hopping rate with Br co-adsorbates divided by the respective rate on the clean surface is plotted vs. the Br chemical potential. The results sensitively depend on the adatom interaction energies from the DFT calculations and therewith on the number of Ag substrate layers used in these calculations. In essence one observes an increase of the S hopping rate with rising Br chemical potential to a maximum of $h \approx 1.6$ to 1.8 at $\mu^{\rm Br} - E^{\rm Br}_{\rm ads} \approx 280$ meV to 320 meV. Within the accuracy of our MC simulation this maximum of h occurs close to or slightly above the order-disorder transition of the Br coverage (compare with Fig. S18a). After reaching the maximum, h rapidly drops to zero with increasing chemical potential, indicating the effect of site blocking by the Br co-adsorbates while approaching the c(2×2) saturation coverage $\theta = 0.5$. Note that our MC model does not account for more complicated $\mathrm{S_{ad}}$ diffusion paths with concerted $\mathrm{S_{ad}}$ and $\mathrm{Br_{ad}}$ movement, which may become relevant at higher coverages. The interaction data in Fig. S16 is converged within 5 meV with respect to the number of Ag substrate layers starting from 14 layers.

In Fig. S18a the Monte Carlo average of the Br coverage θ is shown vs. the Br chemical potential. The MC simulations qualitatively reproduce the well-known increase of the Br coverage with growing Br chemical potential and its saturation at $\langle\theta\rangle = 0.5$[33–37]. Differences are supposed to be due to variations of the interaction energies, as corroborated by additional MC simulations with different choices for the interaction. $\theta_{\rm S} := \langle|\theta_{\rm A} - \theta_{\rm B}|\rangle$ has been suggested by Mitchell et al.[37] as the order parameter for the $p(1 \times 1) \longleftrightarrow c(2 \times 2)$ order–disorder transition. $\theta_{\rm A} = N^{\rm A}_{\rm Br}/(N_{\rm site}/2)$ and $\theta_{\rm B} = (N_{\rm Br} - N^{\rm A}_{\rm Br})/(N_{\rm site}/2)$ denote the Br coverages on the two c(2×2) $\mathrm{Br_{ad}}$ sublattices. The MC average of $|\theta_{\rm A} - \theta_{\rm B}|$ for the $p(6 \times 6)$ simulation cell is shown in Fig. S18b. The non-zero value of $\theta_{\rm S}$ at

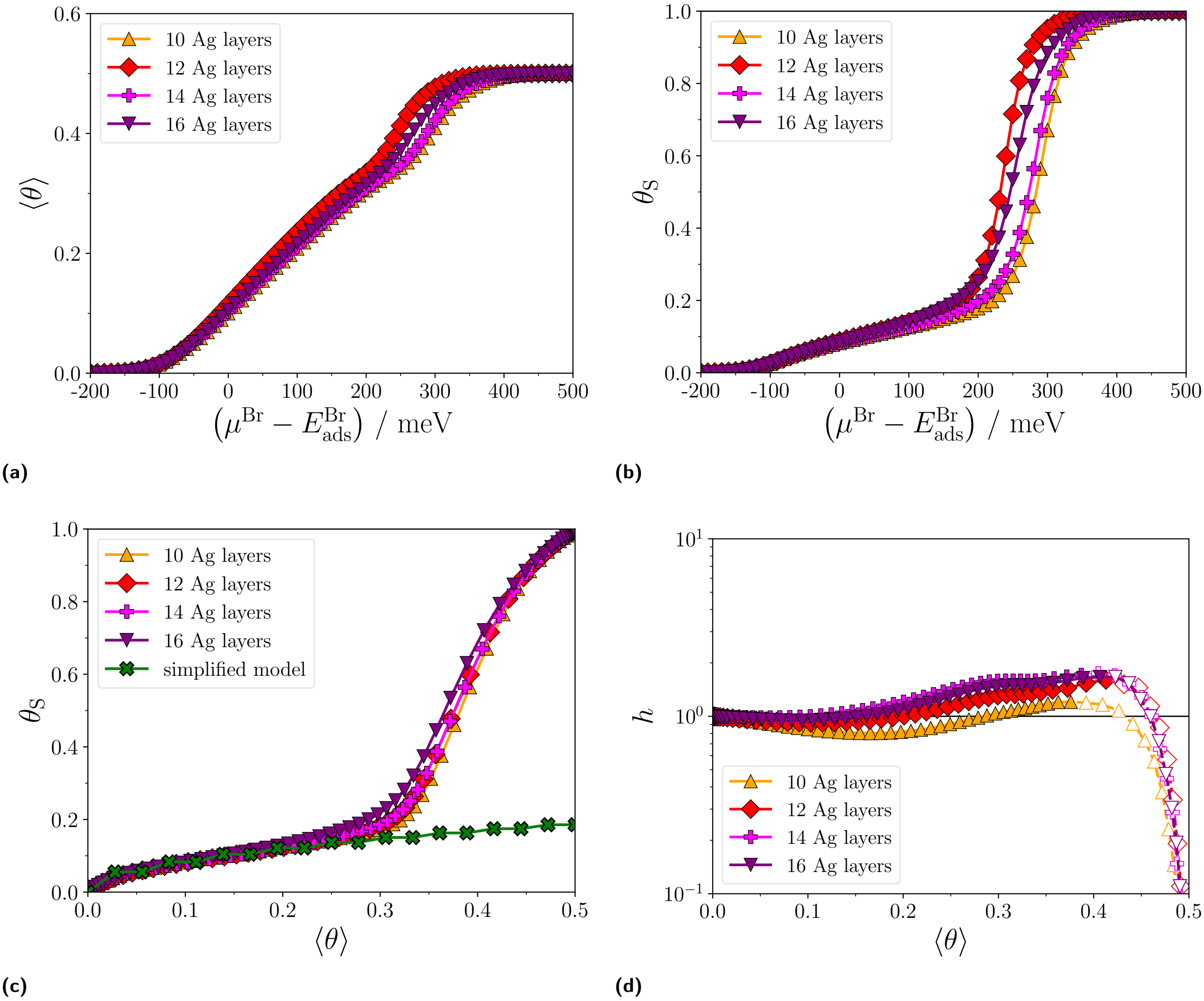

Figure S18. (a) MC results for the Br coverage θ vs. Br chemical potential. (b) MC results for $\theta_S = \langle|\theta_A - \theta_B|\rangle$ vs. chemical potential. (c) Same MC results for θ_S vs. Br coverage compared to data computed from Eq. (15). (d) Same data for the quantity h (Eq. (13)) as in Fig. S17 vs. Br coverage. Lines are guide to the eye.

low Br coverage and the smearing of the order–disorder transition is attributed to finite size effects. The variation of $\langle|\theta_A - \theta_B|\rangle$ can be rationalized with the following rough model: $N_{\rm Br}$ $\rm Br_{ad}$ atoms are randomly distributed with equal probability on the A and B $c(2\times 2)$ sublattices. Br–Br interactions as well as occupation of lattice sites with other $\rm Br_{ad}$ atoms are disregarded. In this case the probability for $N^{\rm A}_{\rm Br}$ atoms on the A lattice is

$$P(N^{\rm A}_{\rm Br}; N_{\rm Br}) = \left(\frac{1}{2}\right)^{N_{\rm Br}} \binom{N_{\rm Br}}{N^{\rm A}_{\rm Br}} . \qquad (14)$$

Within this much simplified approach the expectation value $\langle|\theta_A - \theta_B|\rangle$ calculated in the finite $L \times L$ supercell with $N_{\rm site} = L^2$ sites becomes

$$\sum_{N^{\rm A}_{\rm Br}=0}^{N_{\rm Br}} P(N^{\rm A}_{\rm Br}; N_{\rm Br})|\theta_A - \theta_B| = \left(\frac{1}{2}\right)^{N_{\rm Br}-1} \sum_{N^{\rm A}_{\rm Br}=0}^{N_{\rm Br}} \binom{N_{\rm Br}}{N^{\rm A}_{\rm Br}} \frac{|2N^{\rm A}_{\rm Br} - N_{\rm Br}|}{N_{\rm site}} . \qquad (15)$$

For $L = 6$ this average is displayed as additional green graph in Fig. S18c. It closely follows the MC data for low Br coverage and deviates for higher Br coverage, where the $c(2\times 2)$ Br adlayer reconstruction dominates. The finite value of $\langle|\theta_A - \theta_B|\rangle$ in the $p(1\times 1)$ phase is a finite size effect and vanishes for $L \to \infty$.

We add that in a recent paper N. Bergmann *et al.* have calculated the Br/Ag(100) cyclic voltammogram on the basis of constant potential grand-canonical lattice MC simulations and an implicit solvation model[38].

5. Details on S diffusion via substrate vacancy

As mentioned in the main paper, we have considered an S_{ad} hopping process via a NN Ag vacancy in the first substrate layer. To examine this energetically we have carried out NEB calculations in a p(6 × 6) surface cell with 6 Ag layers for diffusion paths in which the S_{ad} hops from a hollow site into an adjacent Ag vacancy in the presence of different Br co-adsorbate configurations. Examples for initial configurations of such paths with Br_{ad} configurations comprising one Br atom are shown in the insets of Fig. S19a. The final configuration of the paths corresponds to the S-atom residing (subsurface) in the Ag vacancy. Such paths have been calculated for more than 160 Br_{ad} configurations consisting of 1 to 9 Br adatoms, corresponding to Br coverages of $\theta = 1/36$ to $9/36 = 0.25$. The Br_{ad} configurations have been chosen manually with preference for configurations with large S–Br and Br–Br distances to achieve low lateral interaction energy, and therewith a high statistical weight.
For each initial configuration (i.e. S_{ad} next to the Ag vacancy) the Ag vacancy formation energy has been calculated with 8 Ag layers via

$$E^{\text{vac}}_{\text{form}} = E(\text{vac./S/Br/Ag(100)}) + E(\text{Ag bulk}) - E(\text{S/Br/Ag(100)}), \tag{16}$$

where E(vac./S/Br/Ag(100)) and E(S/Br/Ag(100)) are the total energies of the system with or without the Ag vacancy, while E(Ag bulk) is the total energy of one Ag bulk atom. Therewith $E^{\text{vac}}_{\text{form}}$ is the energy cost of creating an Ag vacancy and incorporating the atom into the Ag bulk[39]. For an Ag vacancy in the clean Ag(100) our result of $E^{\text{vac}}_{\text{form}} = 354$ meV compares well to 373 meV from[40] calculated with the same approximation for the XC functional (PBE) and same kind of pseudopotential (PAW).
To compare the statistical significance of the S-into-vacancy hops and the S hollow-bridge-hollow hops, we compare the following two energies

$$E^{\text{S,h-vac}} := \Delta E^{\max}_{\text{NEB}} + E^{\text{vac}}_{\text{form}} + \Delta E^{\text{Br–Br}}_{\text{int,tot}} + \Delta E^{\text{S–Br}}_{\text{int,tot}} \tag{17}$$

and

$$E_{\text{S,h-b-h}} := E^{\text{A,eff}}_{\text{S}} + \langle \Delta E^{\text{Br–Br}}_{\text{int,tot}} \rangle + \langle \Delta E^{\text{S–Br}}_{\text{int,tot}} \rangle. \tag{18}$$

We explain each term. For Eq. (17) we consider a fixed S-Br configuration with an Ag vacancy adjacent to the S adatom:

- $\Delta E^{\max}_{\text{NEB}}$ is the energy barrier of the S-into-vacancy hop calculated by NEB.
- $E^{\text{vac}}_{\text{form}}$ is the Ag vacancy formation energy introduced in Eq. (16).
- $\Delta E^{\text{X–Br}}_{\text{int,tot}}$ is the sum of all X–Br DFT pair interaction energies in the p(6 × 6) cell, X = Br or S, for the considered configuration.

Eq. (18) is to be understood as an averaged quantity over all possible Br configurations in the p(6 × 6) cell, as follows:

- $\langle \Delta E^{\text{X–Br}}_{\text{int,tot}} \rangle$ are θ-dependent MC averages (see Sec. 4.2) for the superposition of S–Br or Br–Br pair interactions.
- $E^{\text{A,eff}}_{\text{S}}$: Proceeding from our MC model for the S hollow-bridge-hollow hopping rate we make the ansatz $\bar{\nu}^{\text{S}}(\theta) =: \nu^{\text{S}}_0 \exp(-\beta E^{\text{A,eff}}_{\text{S}}(\theta))$ to define an effective S hollow-bridge-hollow hop diffusion barrier $E^{\text{A,eff}}_{\text{S}}$ in the presence of Br co-adsorbates with coverage θ. $E^{\text{A,eff}}_{\text{S}}(\theta)$ can readily be computed from the θ-dependent MC results for h in Fig. S18d; we use our most accurate data for 16 Ag layers. Note that this definition implies an approximation: The hopping rate is averaged before taking the logarithm, rather than averaging the energy barriers.

The Ag vacancy concentration in the substrate surface is given by a Boltzmann factor comprising $E^{\text{vac}}_{\text{form}}$[41], and the energy $E^{\text{S,h-vac}}$ in Eq. (17) contains terms that account for this and the adatom interaction energies when deriving the probability of an S-into-vacancy hop for a given S-Br configuration with an NN Ag vacancy next to the S adatom. The energy $E_{\text{S,h-b-h}}$ in Eq. (18) analogously includes an average weight for the S hollow-bridge-hollow hops. However it is not calculated for a fixed S-Br configuration, but rather from MC averages over all Br configurations (without an Ag vacancy) and therefore becomes coverage-dependent.
In Fig. S19a the energy variation along the reaction path from the NEB calculations of the S-into-vacancy hops for all symmetrically inequivalent Br_{ad} configurations with one single Br adatom in the p(6 × 6) cell are plotted exemplarily with an offset determined by the vacancy formation energy plus the lateral interaction energies according to Eq. (17). The maxima of these curves correspond to $E^{\text{S,h-vac}}$ in Eq. (17). For comparison, $E_{\text{S,h-b-h}}$ from Eq. (18) has been added to this Figure as a red horizontal line for a Br coverage $\theta = 1/36 \approx 0.028$ corresponding to Br_{ad} configurations with one single Br_{ad}. By calculating the energy difference

$$\Delta E^{\text{S,h-vac}}_{\text{S,h-b-h}} := E^{\text{S,h-vac}} - E_{\text{S,h-b-h}} \tag{19}$$

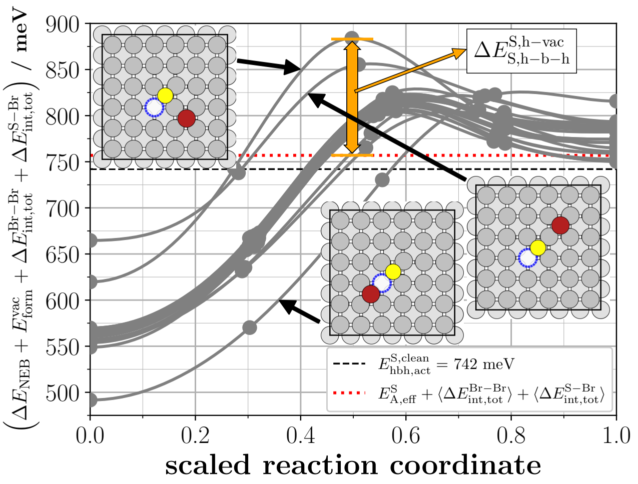

(a)

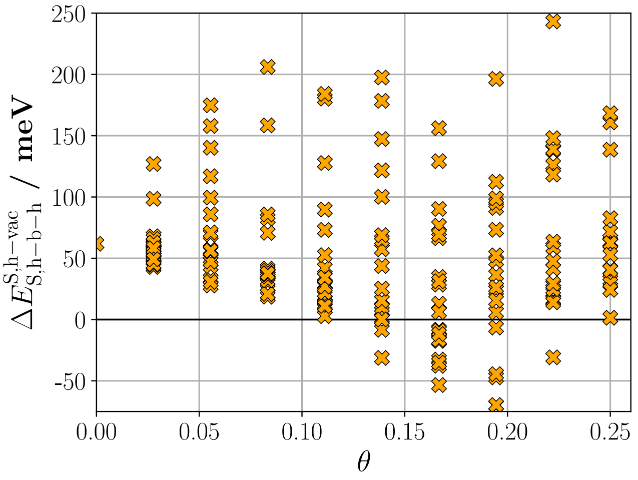

(b)

Figure S19. (a) Exemplary plot of NEB energy change for S-into-vacancy paths (offsetted according to Eq. (17)) vs. a scaled reaction coordinate (0: S_{ad} (yellow) in hollow, 1: S subsurface in adjacent Ag vacancy (blue)). Paths are shown for all symmetrically inequivalent S-Br configurations in the p(6×6) cell consisting of one Br_{ad} (red). The insets show the initial state for some paths. The orange arrow demonstrates the quantity defined in Eq. (19), used to compare the S-into-vacancy and S hollow-bridge-hollow paths energetically (see text for details). (b) Said quantity $\Delta E_{\text{S,h-b-h}}^{\text{S,h-vac}}$ plotted vs. Br coverage for all considered S-Br configurations. For clarity: The $\Delta E_{\text{S,h-b-h}}^{\text{S,h-vac}}$ data from (a) corresponds to the points at $\theta = 1/36 \approx 0.028$ in (b). Note, that for $\theta > 1/36$ not all possible configurations in the p(6×6) have been computed, merely due to their numerousness.

from Eqs. 17 and 18 (see also Fig. S19a), one can estimate whether the S-into-vacancy hop for a certain S-Br configuration is energetically more ($\Delta E_{\text{S,h-b-h}}^{\text{S,h-vac}} < 0$) or less ($\Delta E_{\text{S,h-b-h}}^{\text{S,h-vac}} > 0$) favorable than an (average) S hollow-bridge-hollow hop at the corresponding Br coverage. In Fig. S19b this quantity is plotted vs. Br coverage for all considered S-Br configurations. We note, that the dipole moment difference between transition state and initial state along the S-into-vacancy paths is positive and lies between 79 and 146 meÅ for Br coverage in the range $0 - 0.25$. The energetic difference between the two hopping path alternatives in Fig. S19b appears with both signs. It does not show a clear correlation with θ and amounts in most cases to only a few $k_{\mathrm{B}}T$. This is of similar order of magnitude as the inaccuracy due to the approximation of the XC-functional which is notoriously incorporated in DFT results. Thus, we conclude that no clear trend is revealed, which one of the two suggested hopping processes is energetically preferred in general and that S-via-vacancy hops could also contribute to the S hopping rate measured by experiment.

References

[1] L. Zitzler, B. Gleich, O. Magnussen, R. Behm, *Proc. Electrochem. Soc.* **2000**, *99*, 29.
[2] T. Tansel, O. Magnussen, *Physical Review Letters* **2006**, *96*, 026101.
[3] A. Bewick, B. Thomas, *Journal of Electroanalytical Chemistry and Interfacial Electrochemistry* **1975**, *65*, 911.
[4] A. Taranovskyy, *Study of dynamic processes at the electrochemical interface by in situ high speed STM: Surface diffusion and adsorbate interactions*, Ph.D. thesis, Kiel University **2012**.
[5] T. Tansel, A. Taranovskyy, O. M. Magnussen, *ChemPhysChem* **2010**, *11*, 1438.
[6] M. J. Kochenderfer, T. A. Wheeler, *Algorithms for optimization*, MIT Press **2019**.
[7] G. Ehrlich, F. G. Hudda, *The Journal of Chemical Physics* **1966**, *44*, 1039.
[8] G. Ehrlich, *The Journal of Chemical Physics* **1966**, *44*, 1050.
[9] Y.-C. Yang, O. M. Magnussen, *Physical Chemistry Chemical Physics* **2013**, *15*, 12480.
[10] S. Guezo, A. Taranovskyy, H. Matsushima, O. M. Magnussen, *The Journal of Physical Chemistry C* **2011**, *115*, 19336.
[11] Y.-C. Yang, A. Taranovskyy, O. M. Magnussen, *Langmuir* **2012**, *28*, 14143.
[12] B. Rahn, R. Wen, L. Deuchler, J. Stremme, A. Franke, E. Pehlke, O. M. Magnussen, *Angewandte Chemie International Edition* **2018**, *57*, 6065.
[13] B. Rahn, O. M. Magnussen, *ChemElectroChem* **2018**, *5*, 3073.
[14] J. D. Wrigley, M. E. Twigg, G. Ehrlich, *The Journal of Chemical Physics* **1990**, *93*, 2885.
[15] B. Rahn, O. M. Magnussen, *Journal of the American Chemical Society* **2018**, *140*, 9066.
[16] T. Tansel, A. Taranovskyy, O. M. Magnussen, *ChemPhysChem* **2010**, *11*, 1438.
[17] P. Giannozzi, et al., *Journal of Physics: Condensed Matter* **2009**, *21*, 395502.
[18] P. Giannozzi, et al., *Journal of Physics: Condensed Matter* **2017**, *29*, 465901.
[19] J. P. Perdew, K. Burke, M. Ernzerhof, *Physical Review Letters* **1996**, *77*, 3865.
[20] P. E. Blöchl, *Physical Review B* **1994**, *50*, 17953.
[21] E. Kucukbenli, et al., *arXiv:1404.3015 [cond-mat.mtrl-sci]* **2014**.
[22] H. J. Monkhorst, J. D. Pack, *Physical Review B* **1976**, *13*, 5188.
[23] D. Sheppard, R. Terrell, G. Henkelman, *The Journal of Chemical Physics* **2008**, *128*, 134106.
[24] G. Henkelman, B. P. Uberuaga, H. Jónsson, *The Journal of Chemical Physics* **2000**, *113*, 9901.
[25] S. Smidstrup, A. Pedersen, K. Stokbro, H. Jónsson, *The Journal of Chemical Physics* **2014**, *140*, 214106.
[26] A. H. Larsen, J. J. Mortensen, et al., *Journal of Physics: Condensed Matter* **2017**, *29*, 273002.
[27] T. Juwono, I. A. Hamad, P. A. Rikvold, S. Wang, *Journal of Electroanalytical Chemistry* **2011**, *662*, 130.
[28] S. J. Mitchell, S. Wang, P. A. Rikvold, *Faraday Discussions* **2002**, *121*, 53.
[29] C. R. B. Rodríguez, J. A. Santana, *The Journal of Chemical Physics* **2018**, *149*, 204701.
[30] G. H. Vineyard, *Journal of Physics and Chemistry of Solids* **1957**, *3*, 121.
[31] N. Metropolis, A. W. Rosenbluth, M. N. Rosenbluth, A. H. Teller, E. Teller, *The Journal of Chemical Physics* **1953**, *21*, 1087.
[32] W. H. Press, S. A. Teukolsky, W. T. Vetterling, B. P. Flannery, *Numerical Recipes - The Art of Scientific Computing*, pages 342–343, Cambridge University Press, Cambridge, 3 edition **2007**.
[33] B. Ocko, J. Wang, T. Wandlowski, *Physical Review Letters* **1997**, *79*, 1511.
[34] M. T. Koper, *Journal of Electroanalytical Chemistry* **1998**, *450*, 189.
[35] T. Wandlowski, J. Wang, B. Ocko, *Journal of Electroanalytical Chemistry* **2001**, *500*, 418.
[36] S. Mitchell, G. Brown, P. Rikvold, *Journal of Electroanalytical Chemistry* **2000**, *493*, 68.
[37] S. J. Mitchell, G. Brown, P. A. Rikvold, *Surface Science* **2001**, *471*, 125.
[38] N. Bergmann, N. G. Hörmann, K. Reuter, *Journal of Chemical Theory and Computation* **2023**, *19*, 8815.
[39] D. Mahlberg, A. Groß, *ChemPhysChem* **2021**, *22*, 29.
[40] D.-J. Liu, *Physical Review B* **2010**, *81*, 035415.
[41] Y. Kraftmakher, *Physics Reports* **1998**, *299*, 79.
